# Microbial community structure and composition is associated with host species and sex in *Sigmodon* cotton rats

**DOI:** 10.1186/s42523-021-00090-8

**Published:** 2021-04-16

**Authors:** Britton A. Strickland, Mira C. Patel, Meghan H. Shilts, Helen H. Boone, Arash Kamali, Wei Zhang, Daniel Stylos, Marina S. Boukhvalova, Christian Rosas-Salazar, Shibu Yooseph, Seesandra V. Rajagopala, Jorge C. G. Blanco, Suman R. Das

**Affiliations:** 1 Pathology Microbiology and Immunology, Vanderbilt University Medical Center, Nashville, TN USA; 2 Department of Medicine, Vanderbilt University Medical Center, Nashville, TN USA; 3 Sigmovir Biosystems Inc., 9610 Medical Center Drive, Suite 100, Rockville, MD 20850 USA; 4 Present Address: Influenza Division, National Center for Immunization and Respiratory Diseases, Centers for Disease Control and Prevention, Atlanta, GA USA; 5 Department of Computer Science, Genomics and Bioinformatics Cluster, University of Central Florida, Orlando, FL USA; 6 Department of Otolaryngology, Vanderbilt University Medical Center, Nashville, TN USA; 7 Division of Infectious Diseases, Vanderbilt University Medical Center, 1211 21st Avenue South, S2108 Medical Center North, Nashville, TN 37232 USA

**Keywords:** Microbiome, Metagenomics, Cotton rat, *Sigmodon*, 16S rRNA gene, Gut, Skin, Respiratory

## Abstract

**Background:**

The cotton rat (genus *Sigmodon*) is an essential small animal model for the study of human infectious disease and viral therapeutic development. However, the impact of the host microbiome on infection outcomes has not been explored in this model, partly due to the lack of a comprehensive characterization of microbial communities across different cotton rat species. Understanding the dynamics of their microbiome could significantly help to better understand its role when modeling viral infections in this animal model.

**Results:**

We examined the bacterial communities of the gut and three external sites (skin, ear, and nose) of two inbred species of cotton rats commonly used in research (*S. hispidus* and *S. fulviventer*) by using 16S rRNA gene sequencing, constituting the first comprehensive characterization of the cotton rat microbiome. We showed that *S. fulviventer* maintained higher alpha diversity and richness than *S. hispidus* at external sites (skin, ear, nose), but there were no differentially abundant genera. However, *S. fulviventer* and *S. hispidus* had distinct fecal microbiomes composed of several significantly differentially abundant genera. Whole metagenomic shotgun sequencing of fecal samples identified species-level differences between *S. hispidus* and *S. fulviventer*, as well as different metabolic pathway functions as a result of differential host microbiome contributions. Furthermore, the microbiome composition of the external sites showed significant sex-based differences while fecal communities were not largely different.

**Conclusions:**

Our study shows that host genetic background potentially exerts homeostatic pressures, resulting in distinct microbiomes for two different inbred cotton rat species. Because of the numerous studies that have uncovered strong relationships between host microbiome, viral infection outcomes, and immune responses, our findings represent a strong contribution for understanding the impact of different microbial communities on viral pathogenesis. Furthermore, we provide novel cotton rat microbiome data as a springboard to uncover the full therapeutic potential of the microbiome against viral infections.

**Supplementary Information:**

The online version contains supplementary material available at 10.1186/s42523-021-00090-8.

## Background

The commensal microbiome can dramatically influence many aspects of host health and disease, such as homeostatic signaling, nutrient acquisition, and protection from or exacerbation of infections [1–3]. The majority of early studies established that environmental factors play the major role in shaping and modulating the host microbiome [4–7]. These include factors such as geographic regions and associated cultures and diet in humans [8], and vendor and housing facility in animal model organisms [9, 10]. In addition, recent studies have emphasized that there is a significant role of host genetics on co-evolution of the host and its associated microbiome [11–13]. For example, the murine genetic background is a stronger determinant of microbiome composition and structure than environmental stimuli [14]. Similarly, genetic polymorphisms, heritability, and overall host genetics in humans can also shape how commensal bacteria evolve alongside the host [15–17]. The microbiome has also been instrumental in predicting and protecting against severe viral disease outcomes [18, 19]. However, this burgeoning field of bacteria-host-virus interactions has been limited by a lack of translational models to study mechanisms of virus-microbiome interaction.

Cotton rats (genus *Sigmodon*) are an important small animal model to study various respiratory diseases, including respiratory syncytial virus (RSV) [20], influenza A virus (IAV) [21, 22], parainfluenza virus [23, 24], measles [25], human metapneumovirus [26], enterovirus [27], and human rhinovirus (HRV) [28] due to comparable human disease outcomes [29]. Cotton rats have also provided a useful model for nasal colonization studies (especially with *Staphylococcus aureus*) due to their human-like nasal histology [30]. Furthermore, cotton rats are a useful tool for research since they harbor zoonotic viruses in the wild like Alphavirus (equine encephalitis virus), Hantavirus (Black Creek Canal virus, Bayou virus), Cardiovirus, Arenavirus (Tamiami virus), and Flavivirus (West Nile virus) [31–36].

While mice have been used extensively to study viral immune responses, several factors render the mouse impractical for understanding viral pathology and kinetics, such as those relating to RSV: low replication [not translatable to humans, e.g. RSV replication is 100-fold higher in cotton rats, similar to humans [37]], resistance to upper respiratory infection [unlike humans and cotton rats, RSV does not infect the mouse nasal cavity [38, 39]], divergent lung cell infection [RSV infects ciliated bronchial epithelial cells and alveolar cells in humans and cotton rats, while only infecting pneumocytes in mice [40, 41]], and histological outcomes inconsistent with those similarly seen in the upper and lower airway of both humans and cotton rats [42]. Studies in cotton rats have also accurately predicted efficacy of several RSV therapeutics and vaccines currently used in high-risk human populations [43–46]. In light of all these factors, the cotton rat provides a superior model for studying viral-bacterial interactions than mice.

Furthermore, understanding the cotton rat microbiome is instrumental for understanding microbial interactions with viral infections, as many associational effects of both nasal and gut microbiome composition and modulation of viral outcomes have been well described in mouse models [47–50]. The microbiome of humans [9], mice [10, 51], rats [52], and other animals that are publicly available and used for answering questions relating to host microbiome and disease outcomes have been comprehensively studied and characterized. However, there has not yet been a comprehensive analysis of the microbiome in healthy cotton rats species commonly used in research (i.e., *Sigmodon hispidus* and *S. fulviventer*), making studies of viral-microbiota interactions in this animal model challenging. To date, only one study has examined the nasal microflora of healthy *S. hispidus* but was limited by the sample number and lack of longitudinal timepoints [53].

To comprehensively characterize and establish the structure and composition of the cotton rat microbiome, we collected longitudinal samples from four different body sites of two commonly used inbred cotton rat species, *S hispidus* and *S. fulviventer*, maintained under the same environment and dietary conditions. Our microbiome characterization using both 16S rRNA gene and whole metagenomic sequencing (WMS) comprehensively establishes the microbiome community structure and composition of different body sites in cotton rats and showed distinct community structure based on the cotton rat species and sex. WMS also showed differential metabolic potential of the community between species. Overall, this study not only adds to the small but rapidly expanding literature of the influence of host genetics on the microbiome, but also describes an appropriate animal model for studying microbiome influences on viral and bacterial diseases.

## Results

### Characterization of cotton rat microbiome from multiple body sites

**Fig. 1:**
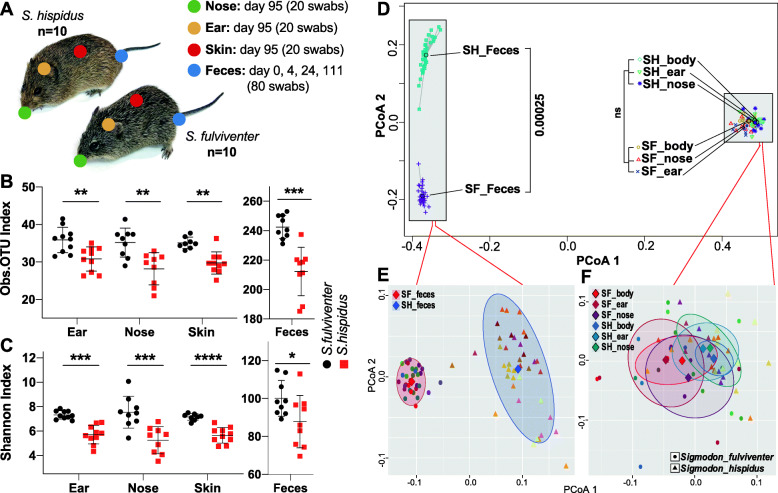
The cotton rat microbiome as examined with 16S rRNA gene sequencing. **a** Study design includes 10 male cotton rats from both *S. fulviventer* and *S. hispidus.* Feces were taken across a 111-day period, while sample swabs of nose, ear, skin were taken at day 95. **b** Site richness (Obs. OTUs) and **c** alpha diversity (Shannon index) metrics at the OTU level indicate that the *S. fulviventer* microbiome was significantly richer and more diverse across all sites. Other alpha-diversity metrics (Chao1, Simpson) are shown in Figure S2. **d** Ordination of samples (Bray-Curtis dissimilarities, OTU level) reveals distinct microbiota compositions between feces and external sites regardless of species. Color and shape of each point indicate the species and body site sampled. **e-f** Zoomed-in beta-diversity relationships between **e** fecal microbiomes and **f** external microbiomes. The color of each point is matched to each individual cotton rat. Statistical testing was performed using PERMANOVA between species. Each site was plotted separately in Figure S3. ns = *P* > 0.05, * = *P* ≤ 0.05, ** = *P* ≤ 0.01, *** = *P* ≤ 0.001, **** = *P* ≤ 0.0001

Two groups of 10 young male cotton rats of *S. fulviventer and S. hispidus* were observed longitudinally for 111 days to characterize the healthy cotton rat microbiome structure and composition. A total of 140 samples were collected and processed for 16S rRNA gene sequencing: ear swabs (20 swabs/day 95), nasal brushes (20 swabs/day 95), skin swabs (20 swabs/day 95), and fecal samples (80 swabs/days 0, 4, 34, and 111) (Fig. 1a). DNA was extracted, and the V4 region of the 16S rRNA gene was amplified then sequenced on the Illumina MiSeq platform with 2 × 250 base pair reads, generating an average of 35,194 reads per sample. Microbiome data was processed by following the mothur SOP, and operational taxonomic units (OTUs) were clustered at 97% identity. For diversity testing, we implemented a sample read cutoff of > 10,000/sample, which utilized 92.9% of samples for analysis with lowest library size of 11,820 reads. Remaining tests to assess differences in abundance of specific taxa (i.e., DESeq2, stabsel, GeneSelector, and LEfSE) used samples passing a per sample read cutoff of > 1000/sample, which utilized 96.4% of samples, with lowest library size of 3678 reads. We then examined the association of alpha and beta diversity (using *vegan* R package) and abundance of taxa (using DESeq2, stabsel, GeneSelector R packages, and LEfSE galaxy portal [54–56]) at each site. To compare community characteristics of cotton rats with other species, we compared beta diversity (Bray-Curtis dissimilarity) between cotton rat fecal samples from Day 0 and both humans from multiple countries [9] and mouse [51] 16S rRNA sequencing data and found that each species had a distinct community composition (Figure S1).

### Differences in the microbiome community structure and composition between cotton rat species

Phyla within *S. fulviventer* and *S. hispidus* external sites (aggregation of ear, nose, and skin samples) were similarly dominated by *Proteobacteria*, *Actinobacteria,* and *Firmicutes*, with only *Tenericutes* being significantly more abundant in *S. fulviventer* (DESeq2 testing; log_2_ fold change = 1.04, q = 3.79E05). Fecal communities consisted mostly of two dominant phyla with opposite abundances between cotton rat species: higher *Bacteroidetes* abundance in *S. fulviventer* compared to *S. hispidus* (50.0% vs. 42.4% respectively, q = 1.69E-06) and higher *Firmicutes* abundance in *S. hispidus* compared to *S. fulviventer* (36.2% vs. 31.8% respectively, q = 1.18E-03) (Table 1). The distribution and differential abundance of top 20 genera in each cotton rat species is shown in Figure S2.

**Table 1:** Percentage abundance of various phyla in both *S.hispidus/S.fulviventer* with standard deviations

Relative abundance ***S.fulviventer/S.hispidus***								
	Body		Ear		Nose		Feces	
Phyla	*S.hispidus*	*S.fulviventer*	*S.hispidus*	*S.fulviventer*	*S.hispidus*	*S.fulviventer*	*S.hispidus*	*S.fulviventer*
*Proteobacteria*	61.1% ± 6.3	56.1% ± 6.0	60.0% ± 9.3	55.7% ± 3.9	63.0% ± 9.9	51.4% ± 8.8	2.4% ± 1.1	2.7% ± 1.0
*Actinobacteria*	21.6% ± 6.8	21.4% ± 5.1	22.5% ± 7.7	19.5% ± 2.8	16.0% ± 3.4	18.5% ± 4.5	0.8% ± 0.5	0.3% ± 0.3
*Tenericutes*	8.6% ± 1.1	11.2% ± 3.0	9.5% ± 3.0	13.9% ± 5.2	13.4% ± 9.5	18.6% ± 13.1	0.3% ± 0.5	2.1% ± 2.8
*Firmicutes*	6.6% ± 1.8	8.9% ± 1.2	6.4% ± 1.7	8.4% ± 1.2	5.8% ± 1.6	9.0% ± 1.8	42.4% ± 9.8	31.8% ± 6.8
*Bacteroidetes*	1.6% ± 0.5	1.9% ± 0.5	1.3% ± 0.4	2.0% ± 0.4	1.4% ± 0.5	1.7% ± 0.5	36.2% ± 9.1	50.0% ± 5.8
other	0.50%	0.50%	0.30%	0.60%	0.30%	0.70%	18.0%	13.2%

We found that the cotton rat gut microbiome was stable over time in both cotton rat species. Richness and alpha diversity did not significantly change over time, no taxa were significantly differentially abundant when experimental day was set as the outcome variable, and beta diversity testing revealed no significant shifts in microbiome composition over time (data not shown). Subsequently, we analyzed groups by computing mean counts for individual cotton rats across time points. Comparison of individual body site microbiomes of both *S. fulviventer* and *S. hispidus* across all time points showed community distinctions between species. All sites (ear, nose, skin, feces) from *S. fulviventer* consistently had higher richness (Observed OTUs, S.chao1 index) and alpha diversity (Shannon Index, Simpson’s Index) when compared to *S. hispidus* (Fig. 1b and c, Figure S3; all values *p* < 0.05). Beta diversity, computed by calculating Bray-Curtis dissimilarity between samples at the OTU level, showed unique composition between the fecal communities of *S. fulviventer* and *S. hispidus* (Fig. 1d; PERMANOVA *p* = 0.00025, beta dispersion *p* = 0.00025, Figures S4, S5D). However, comparison of beta diversity metrics of individual external sites from *S. fulviventer* and *S. hispidus* did not show significant differences (Fig. 1d; Figure S5A-C).

#### Fecal community

**Fig. 2:**
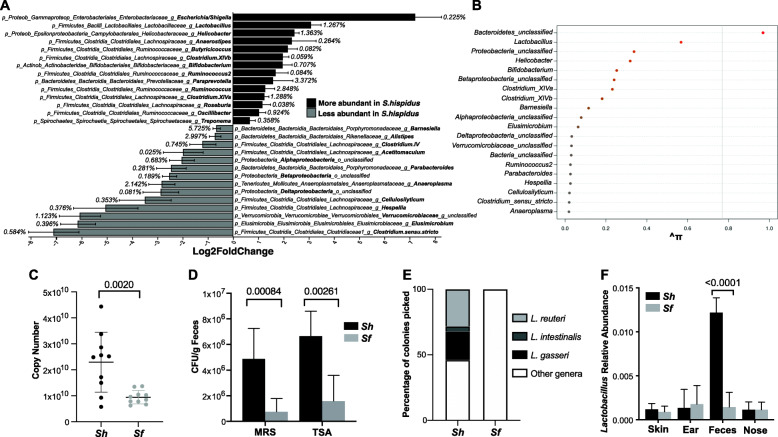
Differential abundance of gut taxa using 16S rRNA gene sequencing. **a** Differential abundance of gut microbial taxa between *S. hispidus* vs. *S. fulviventer* that displayed significant differences (*p* < 0.05, q < 0.05, l2fc > ±0.65) between host species. The Log2FoldChange is plotted along the x-axis, with genera ranked highest in *S. hispidus* (black, +l2fc) to highest in *S. fulviventer* (grey,-l2fc) on the y-axis. Error bars represent the log2 fold change standard error; relative abundances from either *S. hispidus* or *S. fulviventer* are denoted next to the corresponding bar. Unclassified phylogenetic levels indicate the lowest possible classification for that specific OTU. **b** Probability of a gut bacterial genus being selected into a stability selection model distinguishing cotton rat species. The probability of being selected into the model is plotted along the x-axis, with top 20 ranked genera along the y-axis. **c** Bacterial load depicted as copy number/uL of extracted DNA from normalized cotton rat stool. Data were generated by qPCR; and statistics were performed using unpaired T test. **d** Amount of aerobic colony forming units (CFU) per gram of feces on both *Lactobacillus-*selective (MRS) and general growth (TSA) media. *S. hispidus* displayed a higher amount of aerobic growth using both methods. Significance was calculated by the student’s *t*-test. **e** Percentage of CFUs with positive detection of *Lactobacillus* amplicons determined by PCR with primers targeting the *Lactobacillus* 16S rRNA region. Species-specific identity of colonies was confirmed by Sanger sequencing. “Other genera” include *Bacillus*, *Enterococcus*, and *Corynebacterium*. **f** Relative abundance of *Lactobacillus* between cotton rat body sites. Significance was calculated by the student’s *t*-test. In the gut, *Lactobacillus* was one of the higher-abundant taxa with significantly differential abundance between cotton rat species. Figures **c-f** were generated in Prism 8

Analysis using the DESeq2 [55] package identified several bacterial genera that were differentially abundant in the two cotton rat species. These differences were most apparent in the gut (Fig. 2a). *S. hispidus* had a higher abundance of 18 unique genera in the gut (q < 0.05), including *Lactobacillus* (log_2_ fold change = 3.12, q = 3.27E-13), *Helicobacter* (log_2_ fold change = 2.45, q = 2.33E-33), *Anaerostipes* (log_2_ fold change = 2.35, q = 0.029), and *Bifidobacterium* (log_2_ fold change = 1.99, q = 1.03E-06). *Escherichia/Shigella* was more abundant in the *S. hispidus* gut (log_2_ fold change = 7.30, q = 3.79E-11) but had very low relative abundance compared to other genera. *S. fulviventer* had a higher abundance of 18 unique genera in the gut (q < 0.05), including *Clostridium* sensu stricto (log_2_ fold change = − 7.19, q = 7.77E-12), *Elusimicrobium* (log_2_ fold change = − 6.22, q = 8.04E-18), and *Hespellia* (log_2_ fold change = − 5.11, q = 2.21E-04) (Fig. 2a). Full data of differentially abundant taxa at both the genus and family levels are shown in Supplemental File 1. A total of 32 of the 36 DESeq2-calculated differentially abundant genera were also confirmed using the GeneSelector [56] R package (Figure S6, Supplemental File 2). To ensure that no observed differentially abundant taxa were false-positive observations due to low abundances, we utilized the conservative LEfSe test for differential taxa [54], which reported 38 genera and confirmed 35 of 36 DESeq2-calculated differentially abundant genera (except *Clostridia_unclassified*; Supplemental File 3). A stability selection model showed *Lactobacillus* as one of the top genera (as well as those unclassified within the phyla *Bacteroidetes*) with a high probability of predicting whether a fecal sample was from *S. hispidus* or *S. fulviventer* (Fig. 2b). While *Lactobacillus* was one of the top 20 most abundant bacteria of the skin, ear, and nose microbiomes of both *S. fulviventer* and *S. hispidus* (Figure S2), it was not significantly differentially abundant between the two species at any other sites except feces (Fig. 2c).

#### External sites (nose, ear, and skin)

The following sections detail the taxa that were found to be significantly differentially abundant at each site. The full DESeq2 and GeneSelector data at both genus and family levels are shown in Supplementary Files 1 and 2 respectively. LEfSe data at the genus level is shown in Supplementary File 3. All relative abundance values at all phylogenetic levels is shown in Supplementary File 6.

#### Nose community

DESeq2 testing revealed that the *S. hispidus* nose had a higher abundance of *Enterobacteriaceae* (log_2_ fold change = 1.13, q = 1.39E-04) and *Corynebacteriaceae* (log_2_ fold change = 0.445, q = 0.00589), while the *S. fulviventer* nose had a higher abundance of *Leuconostocaceae* (log_2_ fold change = − 1.56, q = 0.064) (Supplementary File 1). LEfSE also revealed that *S. fulviventer* nose had higher abundance of *Facklamia* (LDA = 3.343, *p* = 0.0287), *Bifidobacterium* (LDA = 3.259, *p* = 0.0343), *Turcibacter* (LDA = 3.498, *p* = 0.0161), *Streptococcus* (LDA = 4.023, *p* = 0.00389), and *Actinobacillus* (LDA = 3.969, *p* = 0.00285) (Supplementary File 3).

#### Ear community

By DESeq2 testing, we found that the *S. hispidus* ear had a higher abundance of *Enterobacteriaceae* (log_2_ fold change = 0.48, q = 0.022, confirmed by LEfSe), *Corynebacteriaceae* (log_2_ fold change = 0.571, q = 0.0470), and *Pseudomonas* (log_2_ fold change = 1.33, q = 0.070, confirmed by LEfSe). DESeq2 testing showed that the *S. fulviventer* ear had a higher abundance of *Leptotrichiaceae* (log_2_ fold change = 0.571, q = 0.0470), *Barnesiella* (log_2_ fold change = − 4.58, q = 0.066), and *Porphyromonadaceae* (log_2_ fold change = − 2.11, q = 0.063) (Supplementary File 1). LEfSE confirmed these 3 taxa, as well as higher abundance of *Sphingobacterium* (LDA = 3.299, *p* = 0.00145), *Streptococcus* (LDA = 3.907, *p* = 0.00426), and *Actinobacillus* (LDA = 3.830, *p* = 0.00145) in *S. fulviventer* (Supplementary File 3).

#### Skin community

*Enterobacteriaceae* was more abundant on the *S. hispidus* skin (DESeq2 log_2_ fold change = 0.47, q = 0.0031, confirmed by LEfSe). LEfSe also found more abundant *Streptococcus* (LDA = 3.956, *p* = 0.00769), *Lactococcus* (LDA = 3.494, *p* = 0.0456), *Actinobacillus* (LDA = 3.730, *p* = 0.0129), and *Mycoplasma* (LDA = 4.101, *p* = 0.0330) on the *S. fulviventer* skin. (Supplementary Files 1, 3).

### Confirmation of 16S rRNA gene sequencing data using traditional culture methods

For quantitative comparison of bacterial load between cotton rat species, we used qPCR analysis of total bacterial DNA extracted from homogenized stool (equal weight/volume) and found that the bacterial load was significantly higher in *S. hispidus* than *S. fulviventer* (Fig. 2c). Variance of bacterial load was different between the two species: while all *S. fulviventer* generally had a low bacterial load, the bacterial load had a large range in *S. hispidus*. We also plated an aliquot of normalized, homogenized stool on *Lactobacillus*-specific agar (De Man, Rogosa and Sharpe agar) and observed that the number of colony-forming units (CFU) per gram in *S. hispidus* stool was significantly higher than in *S. fulviventer* stool (Fig. 2d). We found that 86% of colonies picked from *S. hispidus* stool were *Lactobacillus*-positive, compared to zero *Lactobacillus*-positive colonies grown from *S. fulviventer* stool (Fig. 2e). Sequencing of colonies showed *Lactobacillus gasseri* and *Lactobacillus. reuteri* to be the two prominent bacterial species found in *S. hispidus* stool (Fig. 2e). This significant trend supports the relative abundance of *Lactobacillus* as determined by 16S rRNA gene sequencing, where *Lactobacillus* was significantly more abundant in *S. hispidus* compared to *S. fulviventer* (Fig. 2f). Additionally, *Corynebacterium* and *Bacteroides* species were also identified in *S. hispidus* stool samples. Sanger sequencing of isolates from *S. fulviventer* stool identified the presence of *Enterococcus gallinarum* and *E. casselifavus*.

### Differences in the microbiome community structure and composition based on sex

**Fig. 3:**
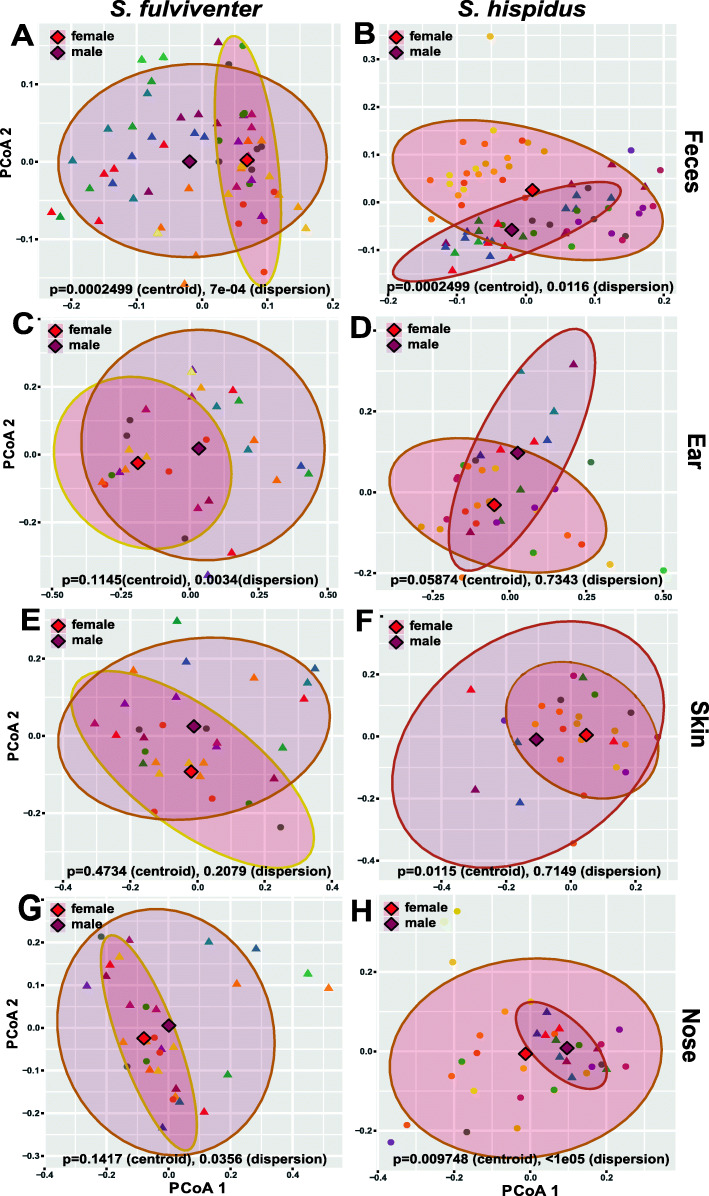
Clustering of site- and species-specific samples (Bray Curtis, OTU level) revealed host sex-dependent communities at most sites. Statistical testing was performed using PERMANOVA for both the geometric mean (or centroid) of the cluster and the dispersion (or variance). **a**, **b** Gut communities showed significant differences between both *S. fulviventer* (SF) and *S. hispidus* (SH) males and females. **c**-**h** External sites (ear, skin, and nose) showed sex-based community trends based on both sample mean distances and dispersion. Longitudinal samples from the same cotton rat are represented by matching point colors

We conducted a secondary analysis to assess if there were any associations between host sex and microbiome community structure and composition. This cohort included 13 *S. fulviventer* cotton rats (10 males, 3 females) and 16 *S. hispidus* cotton rats (5 males, 9 females). Animals in both groups were 4–6 weeks old and weighed approximately 100 g and were observed for 28 days, with fecal samples collected on days 0, 7, 13, 21, and 28 and nose, ear, and skin swabs collected on days 7 and 28. We performed 16S rRNA gene sequencing to examine any effect of host sex. Alpha diversity metrics indicated significant differences in richness (Observed OTUs, Chao1) and diversity (Shannon Index, Simpson Index) between male and female *S. fulviventer* at both the ear and fecal microbiomes (Figure S7A-D), but there were no significant differences in richness and diversity of the microbiomes of male and female cotton rats in the nose and skin for both *S. fulviventer* and *S. hispidus* (with the exception of *S. hispidus* skin diversity, Figure S7D). Overall, differences between host sex were most pronounced in the gut compared to external sites (Figure S7) but only in *S. fulviventer*. Beta-diversity measurements of each species revealed that microbial composition of the gut was significantly dissimilar between male and female cotton rats for both *S. fulviventer* and *S. hispidus* (Fig. 3a, b; *S. hispidus* PERMANOVA *p* = 0.00025, beta-dispersion *p* = 0.1116; *S. fulviventer* PERMANOVA *p* = 0.00025, beta-dispersion *p* = 7E-04). There were also notable differences between male and female at *S. hispidus* skin and nose (Fig. 3e, g). Differential abundance analysis using DESeq2 was conducted between males and females at each site (Table S1). While the fecal community structure differed, there were only 3 differentially abundant genera due to sex in the *S. hispidus* gut and no different genera in *S. fulviventer*. There were differential taxa between sexes at external sites of both *S. hispidus* (21 genera) and *S. fulviventer* (13 genera). Full results at genus and family levels are listed in Supplementary File 4.

### Differences in the microbiome between cotton rat species assessed by whole metagenomic sequencing

Due to the dramatic differences between the *S. fulviventer* and *S. hispidus* gut microbiomes detected by 16S rRNA gene sequencing, we pursued further analysis in order to understand community differences at the species and strain level as well as differences in microbiome functional potential. DNA extracted from 10 male cotton rat stool samples (*S. hispidus* = 5, *S. fulviventer* = 5) at both days 34 and 111 (20 samples total) from the first experimental group were processed for shotgun metagenomic sequencing, which generated 1.11 × 10^9^ reads (5.57 × 10^7^ average reads per sample), comprising of 334,037 megabases (16,701 average megabases per sample) and 17.2% duplicate reads.

**Fig. 4:**
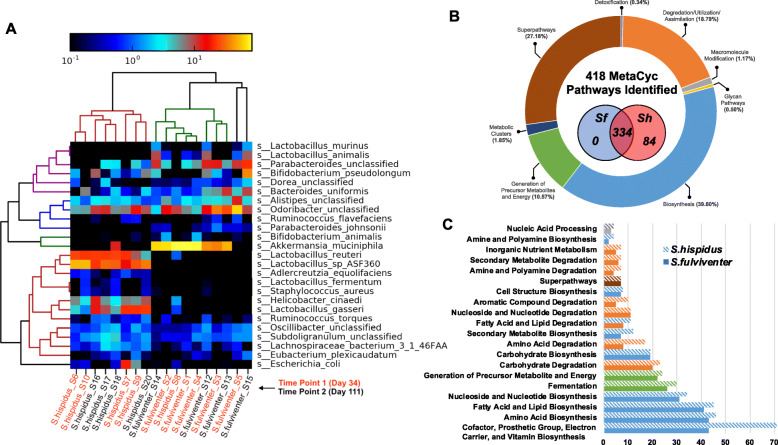
Differential abundance of cotton rat gut taxa and corresponding pathways using whole-genome metagenomic sequencing. **a** Hierarchical clustering (Bray-Curtis) of the top 25 most differentially abundant bacterial species between *S. fulviventer* vs. *S. hispidus*. *Lactobacillus reuteri* and *L. gasseri* were drastically more abundant in *S. hispidus* stool, while *Akkermansia muciniphila* was more abundant in *S. fulviventer* stool. **b** Distribution of MetaCyc metabolomic pathways predicted from bacterial sequences. All 418 unique pathways found were represented in *S. hispidus*, and 334 were shared between *S. hispidus* and *S. fulviventer*. Most pathways were classified within the Biosynthesis, Superpathway, and Degradation/Utilization/Assimilation pathway superclasses. **c** Distribution of pathway ontology for both *S. hispidus* and *S. fulviventer*. Of all the identified pathways, the largest group consisted of Cofactor, Prosthetic Group, Electron Carrier, and Vitamin Biosynthesis, which are often components of host biology sourced solely by commensal bacteria

Whole metagenomic sequencing data showed differences at the species level that validated the 16S rRNA gene sequencing data. Abundances of several bacterial species were found to be statistically different (q < 0.05) between cotton rat species based on taxonomic classification as performed by MetaPhlAn2 followed by differential abundance analysis by both hierarchical clustering (based on Bray-Curtis dissimilarity) of the top 25 most abundant species (Fig. 4a) and DESeq2 (Supplemental File 5). One sample from each *S. fulviventer* and *S. hispidus* were removed from differential expression analysis due to incongruent fitting of hierarchical clustering. *Lactobacillus reuteri*, *L*. *gasseri*, and the novel *L*. sp. ASF360 predominated the gut of *S. hipsidus* (Fig. 4a), and many other *Lactobacillus* species were significantly more abundant in *S. hispidus* samples compared to *S. fulviventer* (Figure S8). Total *Lactobacillus* within the *S. fulviventer* gut was significantly less abundant but included species unique to *S. fulviventer*, including *L. murinus*, *L.* BHWM-4, and *L. animalis*. *Akkermansia muciniphilia* was significantly more abundant in *S. fulviventer* compared to *S. hispidus*. *Ruminococcus torques, Helicobacter cinaedi,* and *Oscillibacter* spp. were of higher abundance in the *S. hispidus* gut. *Parabacteroides* spp. (including *P. johnsonii*) and *Odoribacter* were more abundant in the *S. fulviventer* gut (Supplementary File 5). Proportional counts and raw counts can be found in Supplementary Files 6 and 7 respectively.

### Differential functional potential between cotton rat species microbiome

To understand the biological implications of these differences, HUMAnN2 [57] was used to map any functional differences [MetaCyc pathway database [58]] defined by identified gene families and bacterial profiles. We identified 418 pathways (with nearly all associated with bacteria present in the sample) in the two cotton rat species based on the MetaCyc database, with all 418 pathways represented in *S. hispidus* but only 334 pathways represented in *S. fulviventer* (Fig. 4b). The majority of these pathways included biosynthesis (39.60%) and degradation/utilization/assimilation (18.79%) pathways, as well as several overarching superpathways (27.18%) and energy/metabolite production pathways (10.57%). More specifically, these pathways were part of several instrumental superclass ontologies that metabolize (including de novo pathways) electron carriers, vitamins, fatty acids, lipids, amino acids, carbohydrates, secondary metabolites, and fermentation-derived energy (Fig. 4c). Interestingly, several pathways were differentially abundant between cotton rat species. Each cotton rat species had unique pathways contributed to by their microbiomes (*S. fulviventer* = 14, and *S. hispidus* = 27, *p* < 0.05), and most of these involved biosynthesis (Supplemental File 8).

**Fig. 5:**
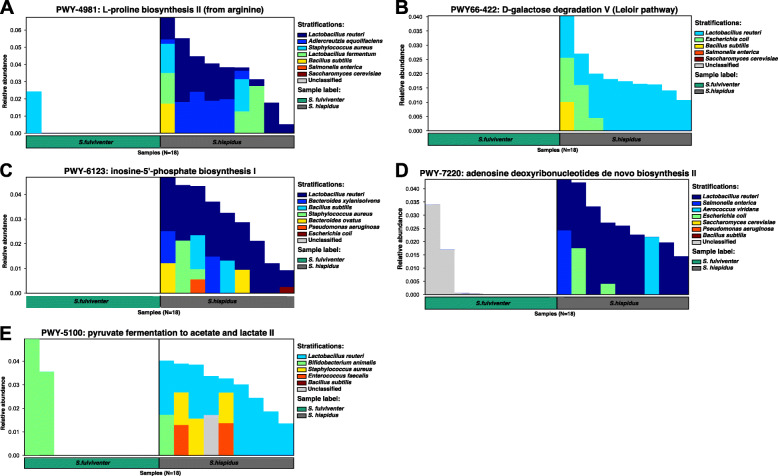
Several pathways that were more active in *S. hispidus* than *S. fulviventer* were greatly contributed to by *Lactobacillus* species. **a** Catalysis of proline biosynthesis by bacterial enzymes (PWY-4981). **b** Catalysis of the conversion of D-galactose to D-glucopyranose 6-phosphate, the more metabolically versatile carbohydrate that can feed directly into glycolysis, by the enzymes of the Leloir pathway (PWY66–422). **c** De novo biosynthesis of purines (PWY-6123). **d** De novo synthesis of ADP for the direct feeding of ATP generation, a pathway that can only accept ribonucleoside diphosphates instead of the mono- or triphosphate forms (PWY-7220). **e** Anaerobic breakdown of glucose to energy (PWY-5100). Table 1 Mean abundance (%) and standard deviation of the most abundant phyla identified with 16S rRNA gene sequencing in both *S.fulviventer/S.hispidus* (listed respectively)

In relation to differentially abundant bacteria species, we found that 44 pathways were solely driven by *Lactobacillus gasseri, L. reuteri*, and L. *ASF360* by matching reads from MetaPhlAn2 bacterial identifications with HUMANn2 predicted pathways. Several of these pathways were more highly expressed in *S. hispidus* (Fig. 5). These included L-proline biosynthesis from arginine (catalyzed by bacterial enzymes), inosine-5′-phosphate biosynthesis (for de novo synthesis of purines), pyruvate fermentation to acetate/lactate (for anaerobic energy production), adenosine deoxyribonuclease de novo biosynthesis (to promote ADP production), and D-galactose degradation (breakdown of D-galactose to a useable form in glycolysis). *Akkermansia municiphilia* was the driver of 25 other pathways, many of which were highly expressed in *S. fulviventer* compared to *S. hispidus* (Figure S9). These included L-isoleucine biosynthesis (for production of leucine and isoleucine), phosphopantothenate biosynthesis (to produce vitamin B5 de novo, of which animals cannot produce, and to feed production of coenzyme A and acyl carrier protein), glycolysis (particularly the degradation of starches for reductants and energy for anabolic pathways), and L-valine biosynthesis. Statistical comparison of all pathways can be found in Supplemental File 9.

## Discussion

Here we have comprehensively characterized the cotton rat microbiome and compared bacterial communities of two different species (*S. hispidus* and *S. fulviventer*) that were housed in the same facility with identical diets. From these analyses, we were able to uncover species-specific differences of gut bacteria, even though the two species had the same diet over many generations and were housed in separate cages in the same room. Interestingly, their external microbiomes (ear, nose, skin) were remarkably similar based on beta-diversity testing and testing for differential bacterial taxa but significantly different based on alpha diversity and richness measurements (which mimicked that of fecal communities). This data further supports that, while environmental factors play a vital role in shaping microbiome structure and composition, underlying host genetics exerts homeostatic pressure for distinct microbiomes between populations.

The most recent phylogenetic analysis of the genus *Sigmodon sp.* found that *S. hispidus* and *S. fulviventer* diverged 5.4 million years ago [59]. In the wild, *S. hispidus* and *S. fulviventer* are sympatric species, with *S. fulviventer* being the more dominant animal [60]. Separate inbreeding of the two species has made them a useful small animal model in laboratory research [29, 61]. Our data show that even when inbred and adapted to a controlled laboratory environment, each species still maintains a unique gut microbiome community structure and composition. The cotton rat is a useful model for respiratory viral infection and therapeutics. In light of recent literature suggesting that the microbiome may play a key role in respiratory viral disease exacerbation or remediation and vaccine response [62–66], our findings exemplify the usefulness of the cotton rat model for understanding viral pathogenesis and treatments in the context of different microbial communities. Furthermore, the cotton rat model could be an optimal subject to uncovering and examining the full therapeutic potential of the microbiome by understanding how the host regulates and modulates bacterial communities.

While other small animal models including mice and rats have been used to explore the relationship between genetic patterns and bacterial homeostasis [14, 67–69], the cotton rat could be used in studying the interplay between differing host microbiomes and host immune responses to viral infections. For example, *S. hispidus* had a significantly higher amount of probiotic gut bacteria genera (*Lactobacillus*, *Bifidobacterium*) that have been associated with protection against the severe outcomes from RSV, IAV, and HRV [50, 70, 71]. Other differentially abundant bacteria between the two species, such as *Escherichia coli* and *Bacillus cereus*, have also been shown to enhance both poliovirus and reovirus replication and pathogenesis [72]. With the presence/absence of these bacteria in this animal model, along with our recently published annotated transcriptome [73], host responses in light of the microbiome could be elucidated. The cotton rat could also be an optimal model for supplementation studies of particular taxa in relation to these viruses.

Here, we have not only comprehensively characterized and established the key differences in microbiome community structure and function between multiple sites from two species of cotton rats housed in same facility for generations and fed with same diet, we also uncovered sex as a factor that can impact the microbiome composition of the gut. In addition, our metagenomic sequencing analysis revealed species level differences as well as the metabolic potential of the microbiome. Differentially abundant taxa were directly related to differentially abundant metabolic pathways between cotton rat species. Pathways such as biosynthesis and degradation pathways (cell structures, electron carriers, vitamins, fatty acids, lipids, amino acids, etc.) could be greatly implicated in mucosal reinforcement that has been previously described in microbiome literature [74]. This microbiome-metabolic characterization may provide an important resource in understanding particular mechanisms by which the microbiome protects against certain disease states.

Our study has significant strengths compared to the lone study published so far [53], including a large sample size, longitudinal sampling, and shotgun metagenomic sequencing to characterize the gut microbiome at the species level. However, we acknowledge several shortcomings: 1) Due to the lack of an assembled cotton rat genome, we could not examine host genes, genetic patterns, or polymorphisms that may be driving differences in microbial colonization. 2) In both cohorts of cotton rats, samples were taken at different time points, and the cotton rats were followed for different durations. However, we found no evidence of changes in the gut microbiome across 111 days of sampling our first cohort of adult cotton rats. 3) We only conducted shotgun metagenomic sequencing on the gut microbiome from male cotton rats from our first cohort. While there is no cotton rat genome in the public databases, with the de novo assembly of the cotton rat transcriptome [73], further studies integrating the microbiome data and gene expression patterns may uncover more relevant information in regard to differences in host and microbiome interactions. We would also like to note that characterizing the microbiome of a unique animal species can be challenging due to the lack of host and/or microbiome sequence databases. The majority of databases and tools are that are commonly available are specifically designed for human microbiome analyses and can result in misclassification of bacteria when used for new animal species. However, until we have better databases, interpretation of data has to be done with caution. Further research is warranted to understand species level microbiome differences and their impact on immune response in all small animal models for better interpretation of preclinical studies of vaccines and anti-microbiological agents. In spite of some limitations, our study creates a steppingstone for future research into these pressing questions of host-microbiome interactions during infection.

## Conclusion

Overall, we have comprehensively characterized the cotton rat microbiome, an invaluable small animal model for viral and bacterial infections, and established key differences in microbiome community structure and function between multiple sites from two species of cotton rats (*S. hispidus* and *S. fulviventer*) housed in same facility for generations and fed with same diet. We also uncovered sex as a variable that can impact the microbiome composition of the gut. This foundational study establishes a platform for future hypothesis testing experiments in understanding the role of microbiome in viral pathogenesis, especially for RSV and Influenza virus. Additionally, this study adds to the small but expanding literature in understanding the role of host genetics on microbiome composition and structure.

## Methods

### Animals

Four- to six-week-old cotton rats (~ 100 g) were obtained from the inbred colony maintained at Sigmovir Biosystems, Inc. (SBI). Cotton rats in the colony were seronegative by ELISA to adventitious respiratory viruses (i.e., Pneumonia Virus of Mice, Rat parvovirus, Rat coronavirus, Sendai virus). Animals were individually housed in large polycarbonate cages and fed a diet of standard rodent chow and water ad libitum.

For rigor and reproducibility, two independent animal experiments were carried out to characterize and establish the healthy cotton rat microbiome structure and composition by comparing two different species, *S. fulviventer and S. hispidus.* In the first experimental group, 20 young male cotton rats were examined: *S. fulviventer* (*n* = 10) and *S. hispidus* (*n* = 10). Each animal was observed for 111 days, with nose, ear, and skin swabs collected at day 95 and fecal samples collected at days 0, 4, 34, and 111. These samples were used for microbiome characterization. To analyze any sex bias to the microbiome, a second experimental group (at a later time) included 13 young *S. fulviventer* (10 males, 3 females) and 16 young *S. hispidus* (5 males, 9 females). Healthy animals were monitored for 28 days with fecal samples collected on days 0, 7, 13, 21, and 28 and nose, ear, and skin swabs collected on days 7 and 28. To avoid fighting, all the animals were housed individually in large polycarbonate cages (with proper enrichment; nylon bone and glass jar). The cotton rat colony was maintained free of human and rodent viruses. All animal procedures followed NIH and USDA guidelines and were approved by the Sigmovir Biosystems, Inc. IACUC.

#### Sample collection

##### Feces collection

One day prior to feces collection, cage beddings were changed in the late afternoon for each animal. Samples were collected between 10 am and 1 pm with sterile forceps. On average, 10–15 feces pellets were collected from each animal. Immediately after collection, samples were frozen at − 80 °C.

##### Nose swab

Sterile saline (~ 100 μl) was pipetted into both nostrils of anesthetized cotton rats positioned face down; Fisherbrand Sterile Swabs (Calcium Alginate Fiber Tipped Ultrafine Aluminum Applicator Swab) were then immediately placed in the nostrils to absorb the saline. Swabs were broken into sterile DNase/RNase-free 1.5 ml tubes and stored at − 80 °C.

##### Ear swab

Sterile saline (~ 100 μl) was pipetted up and down into both ears of each anesthetized cotton rat while the animal was kept in an anesthesia chamber for 1–2 min, and residual liquid was absorbed from each ear with Beaver Visitec Ultracell PVA Eye Spears pack of 5 (intended for fluid absorption and tissue manipulation). Swabs were broken into sterile DNase/RNase-free 1.5 ml tubes and stored at − 80 °C.

##### Skin swab

Sterile saline (~ 200 μl) was put at the back of each anesthetized cotton rat (at approximately the same site for each animal) and rubbed vigorously using Fisherbrand Sterile Swabs (Calcium Alginate Fiber Tipped wood applicator swab). Swabs were broken in a sterile DNase/RNase-free 1.5 ml tubes and stored at − 80 °C.

#### Microbiome DNA extraction and 16S rRNA gene sequencing

Genomic DNA was extracted from all samples at Vanderbilt University Medical Center using the Qiagen DNeasy PowerSoil HTP Kit (96-well plates) following the manufacturer’s protocol, except the optional 4 °C incubations were skipped. Stool samples were thawed on ice and added directly to the kit plate. Nose, ear, and skin swabs were vortexed in tubes with 800 μL Qiagen PowerBead solution for 5 mins; this PowerBead solution was then added to the kit plate. An extraction negative, which did not contain any template but was otherwise processed the same as the rest of the samples, was included on each extraction plate. To mechanically lyse the cells, plates were shaken at 20 Hz in a TissueLyser II system (Qiagen) for 20 min. Steps 16–33 of the kit manufacturer’s protocol were performed on a QIAcube HT (Qiagen). One-step PCR targeting the V4 region of the 16S rRNA gene was performed using 515F/806R primers [75]. MyTaq HS Mix (Bioline) was used to create amplicons, with the following cycling conditions: 95 °C for 2 min; 30 cycles of 95 °C for 20 s, 50 °C for 15 s, 72 °C for 5 min; 72 °C for 10 min; 4 °C indefinitely. Positive PCR results were confirmed by the presence of a 400 bp band in 1% agarose gel electrophoresis; all negative controls were verified at this step to not have a visible band. The PCR products were cleaned and normalized using the SequalPrep Normalization Kit (Invitrogen). Samples and complementary controls (extraction negative, PCR negative, and ZymoBIOMICS Microbial Community Standard) were pooled and then cleaned using 1X AMPure XP beads. Sequencing was done on an Illumina MiSeq platform with 2x250bp reads at the Vanderbilt Technologies for Applied Genomics (VANTAGE) core facility.

#### 16S rRNA gene data processing and statistical analysis

After sequencing, reads were processed using the mothur SOP (https://mothur.org/wiki/miseq_sop/) [76]. Operational taxonomic units (OTUs) were clustered at 97% sequence identity. Non-bacterial sequences, low-quality sequences (1.5% of total reads), and chimeras as identified with UCHIME [77] were removed during data processing. Sequences were taxonomically assigned by the Ribosomal Database Project (RDP) database 14 [78] using the SILVA database release 128 [79]. Samples with < 10,000 final reads (*n* = 10) were removed prior to alpha and beta diversity analysis, and samples with < 1000 final reads (*n* = 5) were removed prior to the remaining analyses to examine specific differentially abundant taxa (i.e., DESeq2, GeneSelector, stability selection, and LEfSE). Statistical analyses were performed using MGSAT [https://bitbucket.org/andreyto/mgsat] [18, 71], which facilitates data analysis by wrapping the R packages as described below.

Alpha- and beta-diversity analyses were performed using the R package *vegan* [80]. Prior to alpha- and beta-diversity analysis, counts were rarefied to the lowest library size, and richness, alpha-, and beta-estimates were calculated. This process was repeated 400 times, and the results were averaged. Richness was estimated with the observed OTUs and Chao1 indices; alpha diversity was estimated with the Shannon and Simpson indices, which were converted into their corresponding Hill numbers [81]. Statistical testing between site alpha diversity was calculated using Mann-Whitney U or Kruskal-Wallace/Dunn’s Post Hoc test where applicable. For beta-diversity analysis, counts were normalized to simple proportions, and pairwise Bray-Curtis dissimilarities were estimated. The PermANOVA (permutation-based analysis of variance) test as implemented in the *Adonis* function from the R package *vegan* was used to test for significance between overall microbial composition and groups of interest (i.e., *S. hispidus* compared to *S. fulviventer* and males compared to females) over 4000 permutations; results are indicated by “centroid” *p*-values. Homogeneity of variance within sample groups was tested using *betadisper* function; results are indicated by “dispersion” *p*-values. Comparisons between *Sigmodon* cotton rats, human, and mouse fecal microbiome communities were performed using the same methods, and data was downloaded from NCBI Short Read Archive database (BioProject PRJNA368790, PRJEB27068, and PRJEB27068). All downloaded data was sampled from a single time point and does not represent longitudinal sampling.

Differential abundance of taxa in association with metadata categories was analyzed using DESeq2 [55] . Prior to DESeq2 analysis, we eliminated all taxa that were had an average number of < 10 reads, taxa with a minimum quantile mean fraction < 0.25, and taxa with a minimum quantile incidence fraction < 0.25; taxa with a normalized base mean (generated by DESeq2) < 10 were removed. Reported adjusted *P* values (q) values are the result of a Wald test with the Benjamini and Hochberg correction to adjust for multiple comparisons. To build alternative rankings of taxa in regard to their prevalence in one cotton rat species over the other, we also used stabsel and GeneSelector. The stabsel stability selection [82] approach aims to build the relative ranking of the predictor variables (taxa in this case) according to their importance for predicting the outcome. It does so by building multiple “base” models on random subsamples of the data. The elastic net model from the R package glmnet was used as the base feature selection method to be wrapped by the stability protocol. The ranking of taxa and their probability of being selected into the model were reported, as well as the probability cutoff corresponding to the per-family error rate that is controlled by this method. The GeneSelector package [56] was used as a stability feature ranking method that is based on a nonparametric univariate test. In brief, the same ranking method (package function RankingWilcoxon) was applied to multiple random subsamples of the full set of observations (400 replicates, sampling 50% of observations without replacement). RankingWilcoxon ranks features in each replicate according to the test statistic from Wilcoxon rank-sum test with regard to the outcome group (e.g. *S. hispidus* vs. *S. fulviventer*). Consensus ranking between replicates was then found with a Monte Carlo procedure (package function AggregateMC) and the features were reported in the order of that consensus. To account for different sequencing depth, the absolute abundance counts were normalized to simple proportions within each observation. For each feature, we also obtained several types of the effect size, such as common language effect size and rank biserial correlation. LEfSe (Linear discriminant analysis [LDA] Effect Size) was executed using the online Galaxy module [54] to determine taxa most likely to explain differences between classes (species, sex, etc) using feature ranking followed by Kruskall-Wallis and pairwise Wilcoxon tests. These 4 statistical analyses (DESeq2, stabsel, GeneSelector, and LEfSe) allowed for rigorous testing of each particular taxon of interest.

#### Metagenomic library preparation

A subset of fecal samples from 20 total male cotton rats (10 from each species), taken at days 34 and 111 within the first cohort of cotton rats, underwent whole-metagenomic shotgun sequencing. From the same stool samples, genomic DNA was extracted using the Qiagen DNeasy PowerSoil Kit (Cat No./ID: 12888–100) by following the manufacturer’s protocol (skipping the optional 4 °C incubations). In addition, a negative sample (which did not contain any template but was otherwise processed the same as the rest of the samples) and a positive control (ZymoBIOMICS Microbial Community Standard) were processed in parallel with samples and sequenced. Samples were normalized to 75 ng/ μL in 1X TE prior to library construction. Metagenomic libraries were prepared using the NEBNext® Ultra™ II FS DNA Library Prep Kit for Illumina® following the manufacturer’s protocol for inputs ≤100 ng. Samples were fragmented at 37 °C for 12 min to yield a fragment size of 200–450 bp. NEBNext Multiplex Adaptors were diluted 10-fold. NEBNext Multiplex Oligos for Illumina (Set 1, NEB #E7335) were used for PCR enrichment of adaptor-ligated DNA, and 5 cycles of PCR were run. Library quality was assessed on an Agilent 2100 Bioanalyzer System using the Agilent High Sensitivity DNA Kit (5067–4626). Samples were sequenced via the NovaSeq 6000 2 × 150 platform for Illumina at the Vanderbilt Technologies for Advanced Genomics (VANTAGE) core, aiming for 40 million reads per sample.

#### Whole metagenomic shotgun sequence analysis

FastQC [83] followed by MultiQC [84] were used to examine data quality. Trimmomatic [85] was used to remove adaptors and trim low quality reads using the parameters: TRAILING:3 SLIDINGWINDOW:4:15 MINLEN:75. An average of 85% of reads mapped to various host DNA databases, but reads were not filtered before functional classification. Microbial communities were then profiled using MetaPhlAn2 [86] . Differentially abundant bacteria were calculated using MetaPhlAn2’s *hclust2.py* function by hierarchical clustering (based on Bray-Curtis dissimilarity) of the top 25 most abundant species according to the 90th percentile of the abundance in each clade as well as DESeq2. Functional, metabolic profiles were analyzed using HUMANn2, which aligns reads from UniRef [87] and clusters abundances to the ChocoPhlAn [57] database. This generates three outputs: UniRef IDs for gene families in reads per million, MetaCyc pathway coverage, and MetaCyc pathway abundances in copies per million (Supplemental File 9). To identify differential pathways between sample groups, associations between cotton rat species were identified by the HUMANn2.associate script and statistical testing using the Kruskal-Wallis H-test. Data presented (generated by HUMANn2.barplot script) is from pathway abundances (normalized as relative abundance) within each sample with unmapped/unintegrated pathways removed and was found statistically significant (*p* < 0.05 and q < 0.05). Superclasses distribution of identified MetaCyc pathways was manually generated using the online MetaCyc database.

#### Enumeration of Lactobacillus

Two frozen stool pellets were taken from 20 male cotton rats (10 *S. hispidus*, 10 *S. fulviventer*), weighed, and diluted to 45 mg/mL in sterile 1x PBS. Samples were rocked for 20 min on ice and resuspended manually with pipette mixing. 10^− 1^–10^− 3^ serial dilutions were plated on *Lactobacilli* MRS agar (BD 288210) and incubated at 37 °C for 48 h. Colonies on 10^− 2^ were counted, and 95 colonies were randomly picked from each species and inoculated into 1.2 mL MRS broth (BD 288130) in a sterile 96-deep-well plate. The plate was incubated at 37 °C for 20 h with no shaking. Cultures were gently mixed by pipetting, and 20% glycerol stocks were prepared for each culture. Colony PCR was performed on each isolate by boiling the culture at 95 °C for 10 min, then using 10 μL as the template with *Lactobacillus* species-specific primers [88] and MyTaq HS Red (Bioline®) with the following cycling conditions: 95 °C for 2 min; 30 cycles of 95 °C for 20 s, 50 °C for 15 s, 72 °C for 1 min; 72 °C for 10 min; 4 °C indefinitely. PCR reactions were spun at 3900 g for 10 min to remove any bacterial debris from the boiled template and run on 1% agarose gel to verify *Lactobacillus*-positive colonies. A new PCR was then repeated using the universal primers Uni331F/Uni797R [89] (following cycling conditions listed above), and purified PCR products were sent for Sanger sequencing. Bacterial isolate identity was determined using NCBI BLAST database.

#### Determination of bacterial load by qPCR

DNA was extracted from an equal volume of normalized homogenates of cotton rat stool (described in Methods: Enumeration of *Lactobacillus*) using the DNeasy PowerSoil Kit (Qiagen). qPCR reactions were prepared in duplicate using BioRad iQ Supermix with Invitrogen Sybr Green following the manufacture’s protocol. Universal eubacteria 16S rRNA primers (UniF340, UniR514) [90] equal volumes of extracted DNA, and targeted standards were used to determine copy number per gram of feces. Each qPCR plate included a corresponding extraction negative and a no-template negative control. A serial dilution of standards containing known bacterial copy numbers specific to the primer pair were used as a standard curve as previously described. PCR reactions were run with a 15 s 95 °C melting and 1 min 54 °C annealing step for 40 cycles. Cycle threshold (CT) values were plotted against the standard curve to determine copy number, and figures and statistical testing (unpaired T test) were generated using Prism version 8.

#### Isolation and culture of Lactobacillus strains from cotton rats’ stool

Glycerol stocks of identified *Lactobacillus* species were streaked on MRS agar plates, and a single colony was grown in culture using MRS broth. To determine growth parameters, each species was incubated at 37 °C without shaking, and growth efficiency was measured by turbidity. A growth curve was also estimated using a BioTek Synergy HTX plate reader at 37 °C for 24 h; OD_600_ was measured every 10 min following a brief 3 s shake to mix culture. CFU counts were also taken during the log phase by plating a 3-fold serial dilution on MRS agar plates.

## Supplementary Information

**Additional file 1: Figure S1.** Ordination of human, mouse, and two *Sigmodon* cotton rat species (Bray-Curtis dissimilarities, OTU level) reveals that the cotton rat fecal microbiome is very distinct from that of humans, and more similar to, but still distinct from, mice.

**Additional file 2: Figure S2.** Top 20 most abundant bacterial genera at each body site of *S. hispidus* and *S. fulviventer.* In both species of cotton rats, external sites (skin, ear, nose) shared similar dominating genera while there were notable difference in gut taxa between *S. hispidus* and *S. fulviventer*. Not all reads were able to be classified down to the genus level; the lowest taxonomic level available is reported. The letter after the classification denotes the lowest taxonomic level able to be identified for the particular OTU (i.e., g for genus, f for family, o for order, c for class, p for phylum).

**Additional file 3: Figure S3.** Chao1 and Simpson alpha diversity indices of ear, nose, skin, and feces between *S. fulviventer* and *S. hispidus*. Statistical testing between each cotton rat species was performed using a Student’s *t*-test. Statistical testing between body sites is not shown (no significant differences across external sites). ns = *P* > 0.05, * = *P* ≤ 0.05, ** = *P* ≤ 0.01, *** = *P* ≤ 0.001, **** = *P* ≤ 0.0001.

**Additional file 4: Figure S4.** PCoA plots comparing Bray-Curtis dissimilarities between body sites in both (A) *S. hispidus* and (B) *S. fulviventer.*

**Additional file 5: Figure S5.** Difference in body site beta diversity between *S. hispidus* and *S. fulviventer*. Clustering of samples (Bray-Curtis, OTU level) shows separation by host species (A) Ear, (B) Skin, (C) Nose, (D) Feces.

**Additional file 6: Figure S6.** Statistically significant differentially abundant bacteria genera between *S. hispidus* and *S. fulviventer* determined by GeneSelector.

**Additional file 7: Figure S7.** Alpha diversity metrics of ear, nose, skin, and feces between male and female *S. fulviventer* and *S. hispidus*. Richness and diversity were determined using the following methods: A) Observed OTUs, B) Chao1 index, C) Shannon Diversity Index, and D) Simpson Diversity Index. Statistical testing between gender of each cotton rat species was performed using a one-way (feces) and two-way (external sites) ANOVA, followed by a Tukey’s post-hoc test. The *p*-value as a result of all comparisons is shown in the top right; pairwise comparisons that were found to be significant with the Tukey post-hoc test are denoted by a bar and asterisk above the groups being compared. Statistical testing across species is not shown. Feces were plotted separately to account for the discrepancy between the Y axes. ns = *P* > 0.05, * = *P* ≤ 0.05, ** = *P* ≤ 0.01, *** = *P* ≤ 0.001, **** = *P* ≤ 0.0001.

**Additional file 8: Figure S8.** DESeq2 results representing differential abundance of *Lactobacillus* species and strains between *S. hispidus* and *S. fulviventer.* Data were generated from whole metagenome sequencing.

**Additional file 9: Figure S9.** Several pathways that were more active in *S. fulviventer* than *S. hispidus* were greatly contributed to by *Akkermansia* species. (A) A member of the superpathway of branched chain amino acid biosynthesis, that generates not only isoleucine, but also leucine and valine (ILEUSYN-PWY). (B) Degradation of starch for the generation of carbon skeletons, reductants, and ATP for anabolic bacterial fatty acid pathway initiation via pyruvate decarboxylation to acetyl CoA (PWY-1042). (C) Biosynthesis of R-4′-phosphopantothenate, the universal precursor for the synthesis of coenzyme A and acyl carrier protein. Only plants and microorganisms can synthesize pantothenate de novo; animals require a dietary supplement. Synonymous with Vitamin B5 synthesis. (PANTO-PWY). (D) A member of the superpathway of branched chain amino acid biosynthesis, that generates not only valine, but also leucine and isoleucine.

**Additional file 10: Table S1.** Differential abundance analysis of taxa between individual body site across female and male *S. hispidus* and *S. fulviventer*. Positive Log2FoldChange = higher in females; negative Log2FoldChange higher in males. There were no significant taxa in *S. fulviventer* feces.

**Additional file 11: Supplemental File 1.** DESeq2 analysis of 16S rRNA gene sequencing data between *S. fulviventer* and *S. hispidus* across all body sites at the genus and family level.

**Additional file 12: Supplemental File 2.** GeneSelector analysis of 16S rRNA gene sequencing data between *S. fulviventer* and *S. hispidus* across all body sites at the genus and family level.

**Additional file 13: Supplemental File 3.** LEfSe analysis of 16S rRNA gene sequencing data between *S. fulviventer* and *S. hispidus* across all body sites at the genus level.

**Additional file 14: Supplemental File 4.** DESeq2 analysis of 16S rRNA gene sequencing data between male and female cotton rats (both *S. fulviventer* and *S. hispidus*) across all body sites at the genus and family level.

**Additional file 15: Supplemental File 5.** DESeq2 analysis of whole metagenomic sequencing data between *S. fulviventer* and *S. hispidus* across all body sites at the species level.

**Additional file 16: Supplemental File 6.** Proportional counts from 16S rRNA sequencing data at all levels for both cotton rat cohorts.

**Additional file 17: Supplemental File 7.** Raw counts from 16S rRNA sequencing data at all levels for both cotton rat cohorts.

**Additional file 18: Supplemental File 8.** All annotated MetaCyc pathways identified by HUMANn2 from whole metagenomic sequencing data.

**Additional file 19: Supplemental File 9.** Statistical comparison of MetaCyc pathway comparison between *S. fulviventer* and *S. hispidus*.

## Data Availability

.Sequencing data is availble through the NCBI Short Read Archive (SRA) under BioProject PRJNA721429. Additionally, please contact corresponding authors for data requests.

## Abbreviations

RSV: Respiratory Syncytial Virus
Influenza A Virus: Influenza A Virus
HRV: Human Rhinovirus
rRNA: Ribosomal Ribonucleic Acid
WMS: Whole Metagenomic Sequencing
OTU: Operational Taxonomic Unit
LDA: Linear Discriminant Analysis
LEFSE: Linear Discriminant Analysis [LDA] Effect Size
CFU: Colony-Forming Units
RT-QPCR: Reverse Transcription Quantitative Polymerase Chain Reaction
