# Supplementary figures and images for: Microbial community structure and composition is associated with host species and sex in Sigmodon cotton rats
Source: Anim Microbiome. 2021 Apr 16;3:29. doi: 10.1186/s42523-021-00090-8 (PMC8051552; doi:10.1186/s42523-021-00090-8)

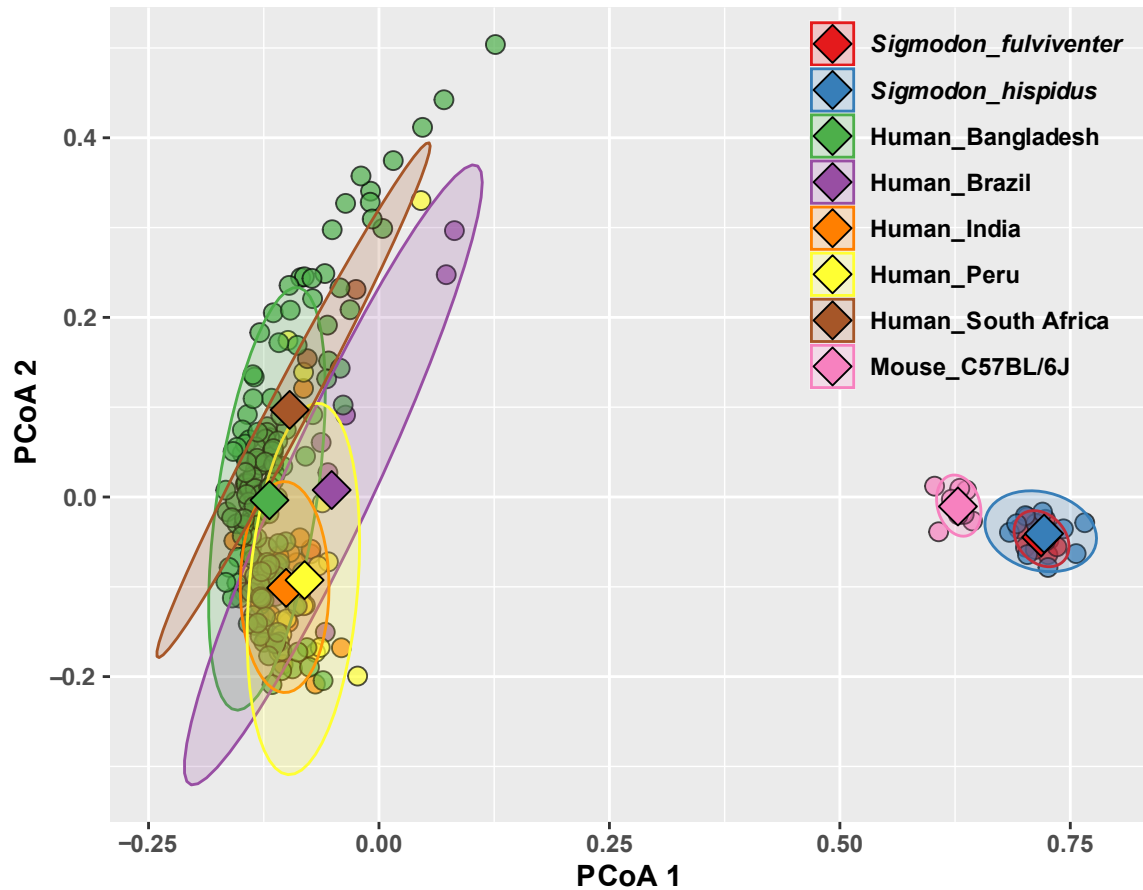

Supplement: Supplementary file 1 — Additional file 1: Figure S1. Ordination of human, mouse, and two Sigmodon cotton rat species (Bray-Curtis dissimilarities, OTU level) reveals that the cotton rat fecal microbiome is very distinct from that of humans, and more similar to, but still distinct from, mice. [file 42523_2021_90_MOESM1_ESM.pdf]

## Chao1 Index

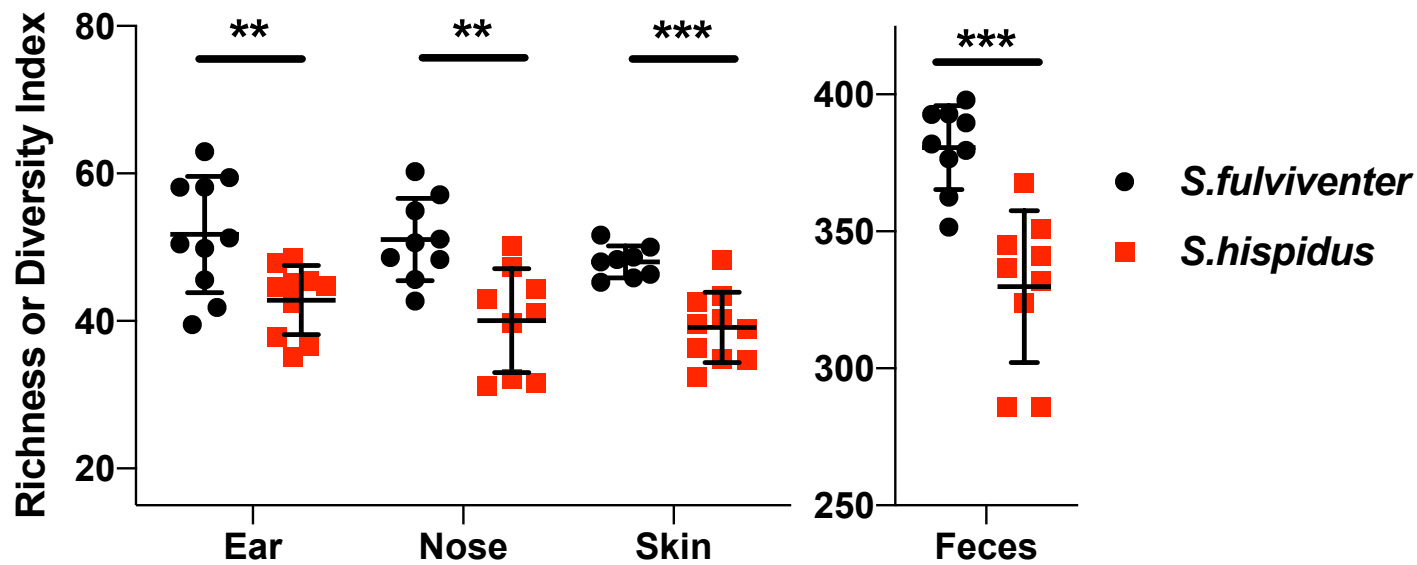

## Simpson Diversity Index

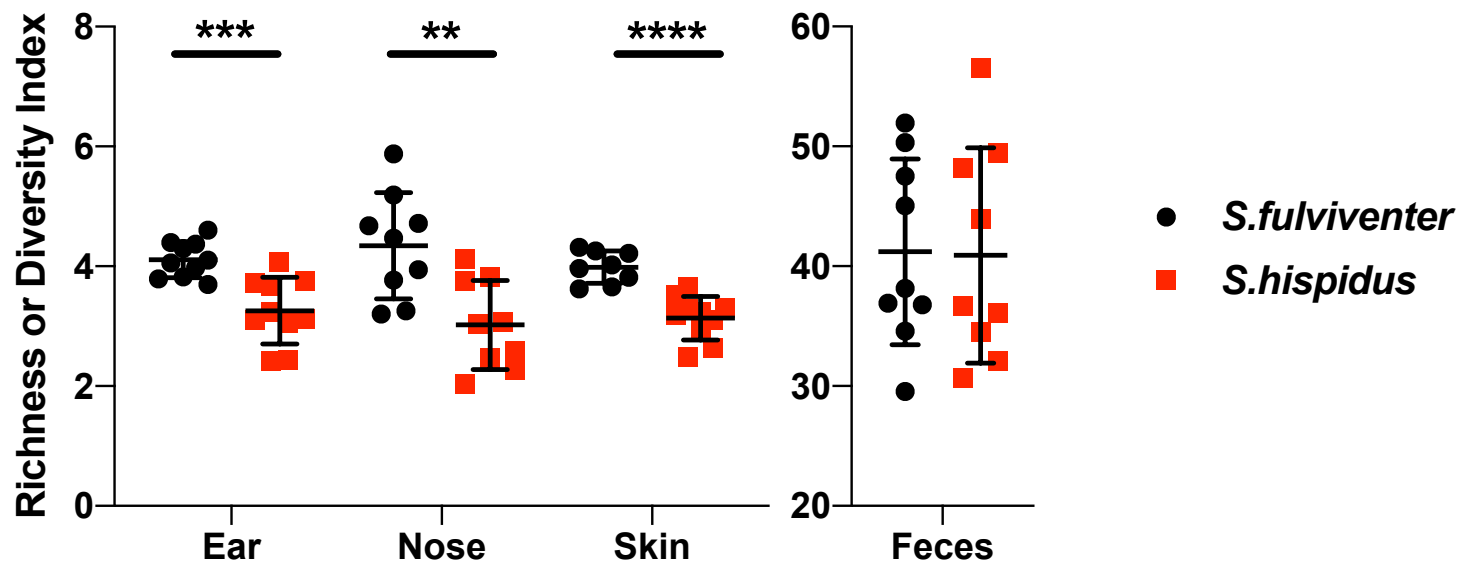

Supplement: Supplementary file 3 — Additional file 3: Figure S3. Chao1 and Simpson alpha diversity indices of ear, nose, skin, and feces between S. fulviventer and S. hispidus. Statistical testing between each cotton rat species was performed using a Student’s t-test. Statistical testing between body sites is not shown (no significant differences across external sites). ns = P > 0.05, * = P ≤ 0.05, ** = P ≤ 0.01, *** = P ≤ 0.001, **** = P ≤ 0.0001. [file 42523_2021_90_MOESM3_ESM.pdf]

**A**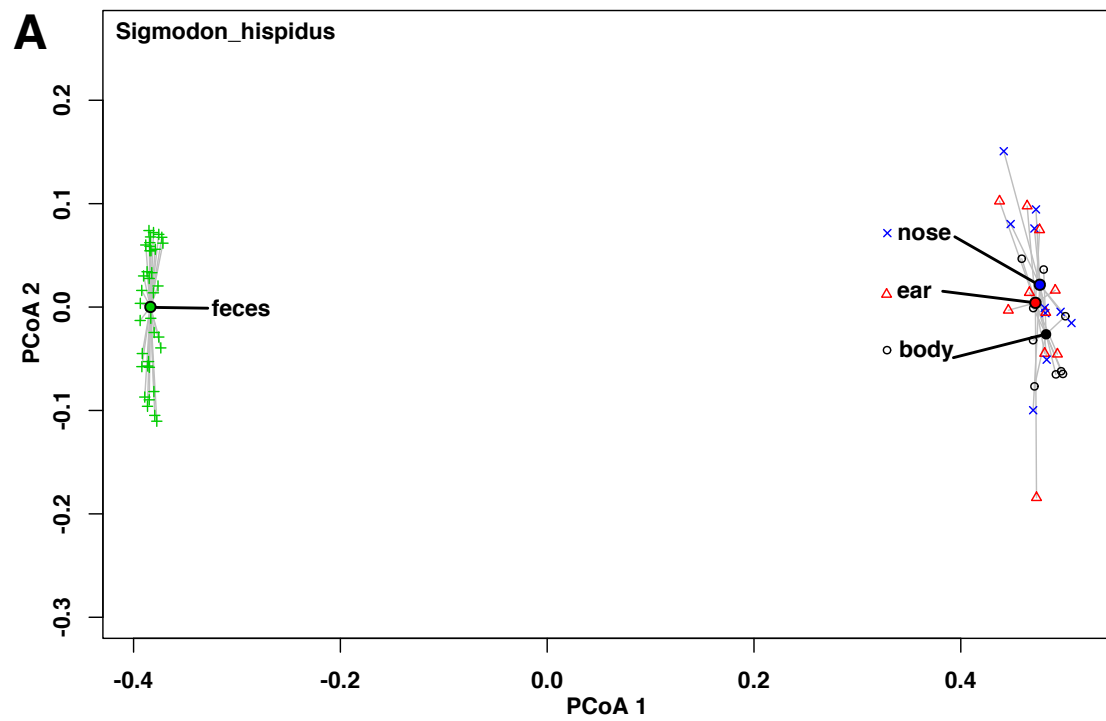**B**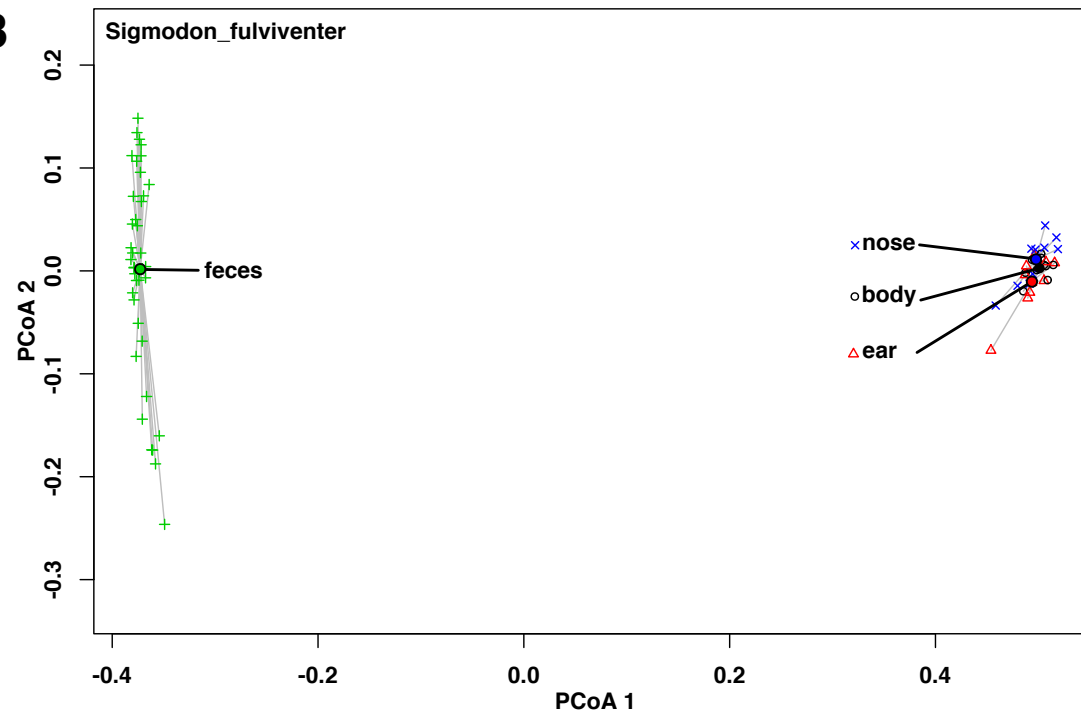

Supplement: Supplementary file 4 — Additional file 4: Figure S4. PCoA plots comparing Bray-Curtis dissimilarities between body sites in both (A) S. hispidus and (B) S. fulviventer. [file 42523_2021_90_MOESM4_ESM.pdf]

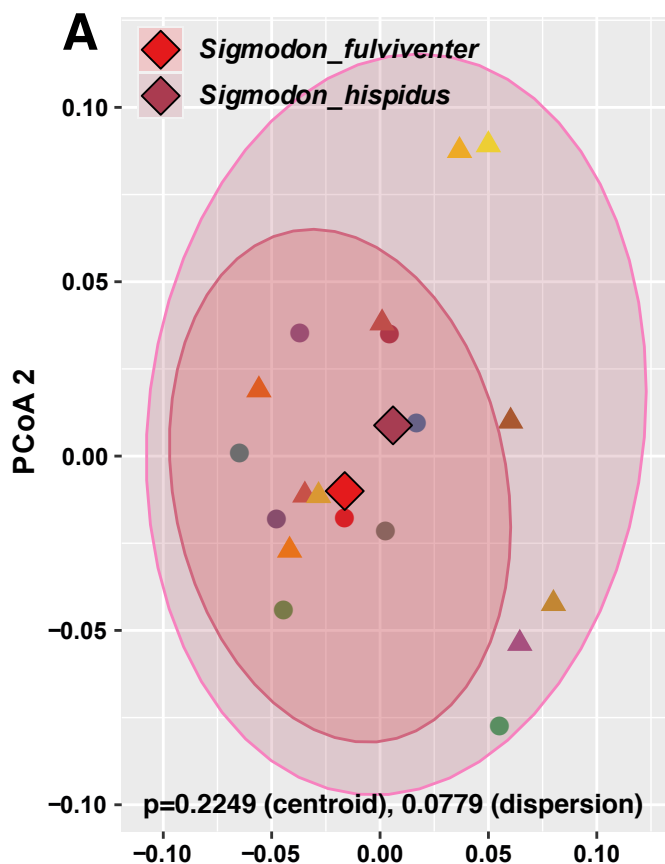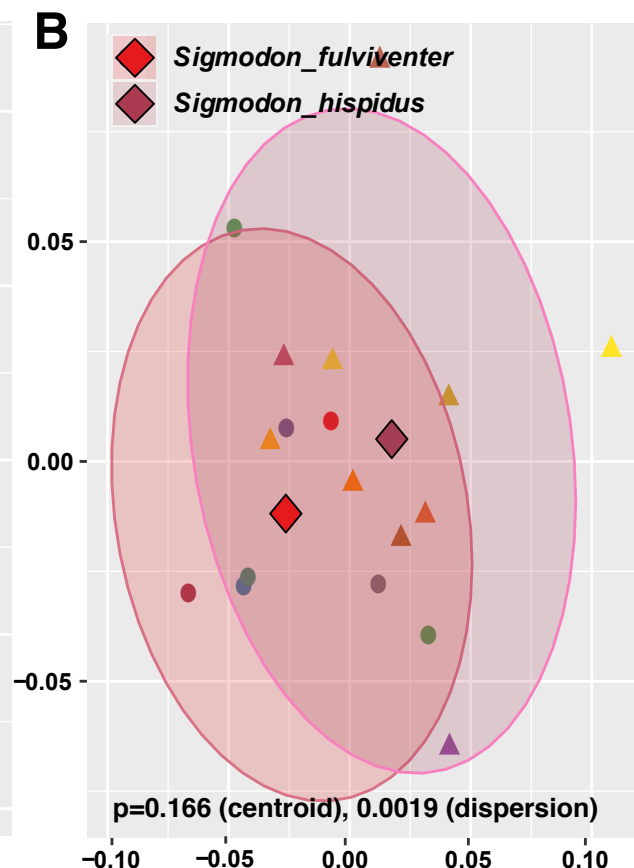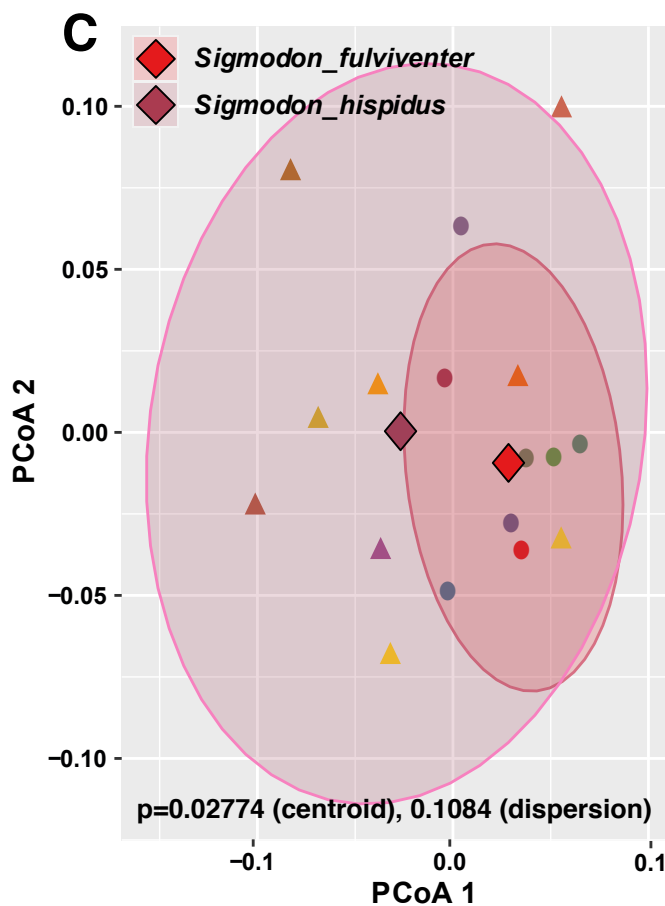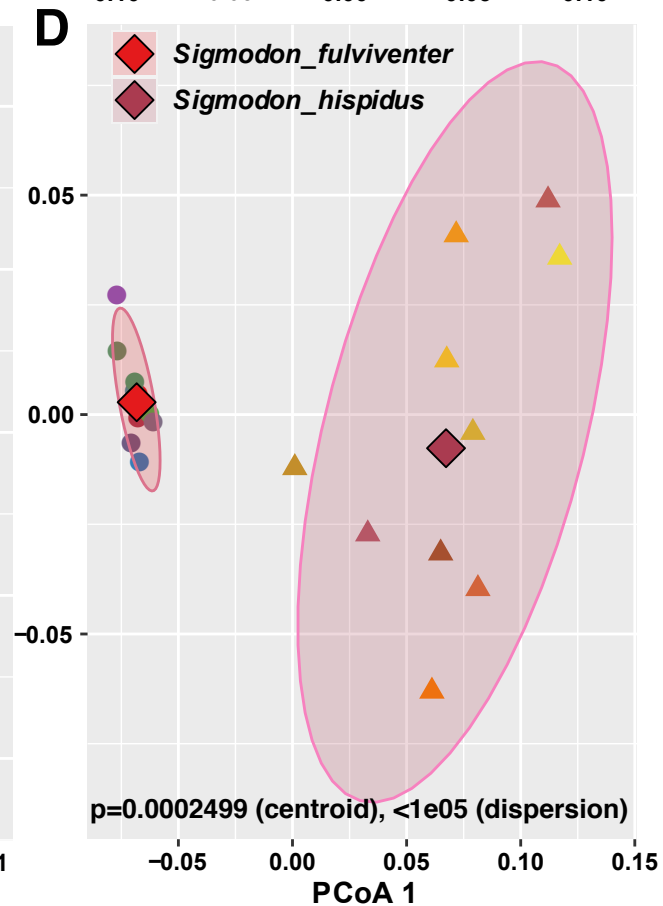

Supplement: Supplementary file 5 — Additional file 5: Figure S5. Difference in body site beta diversity between S. hispidus and S. fulviventer. Clustering of samples (Bray-Curtis, OTU level) shows separation by host species (A) Ear, (B) Skin, (C) Nose, (D) Feces. [file 42523_2021_90_MOESM5_ESM.pdf]

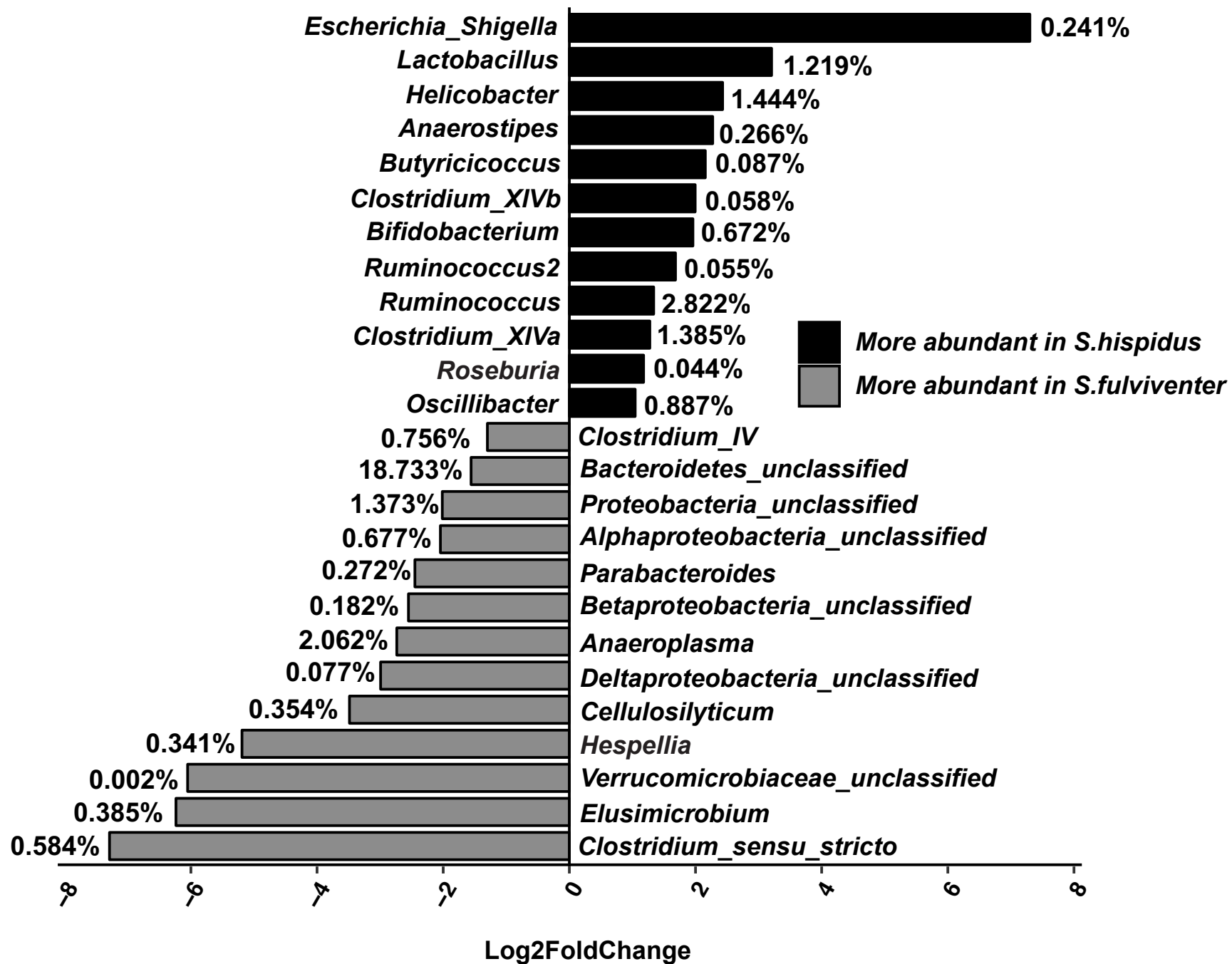

Supplement: Supplementary file 6 — Additional file 6: Figure S6. Statistically significant differentially abundant bacteria genera between S. hispidus and S. fulviventer determined by GeneSelector. [file 42523_2021_90_MOESM6_ESM.pdf]

FIGURE S6

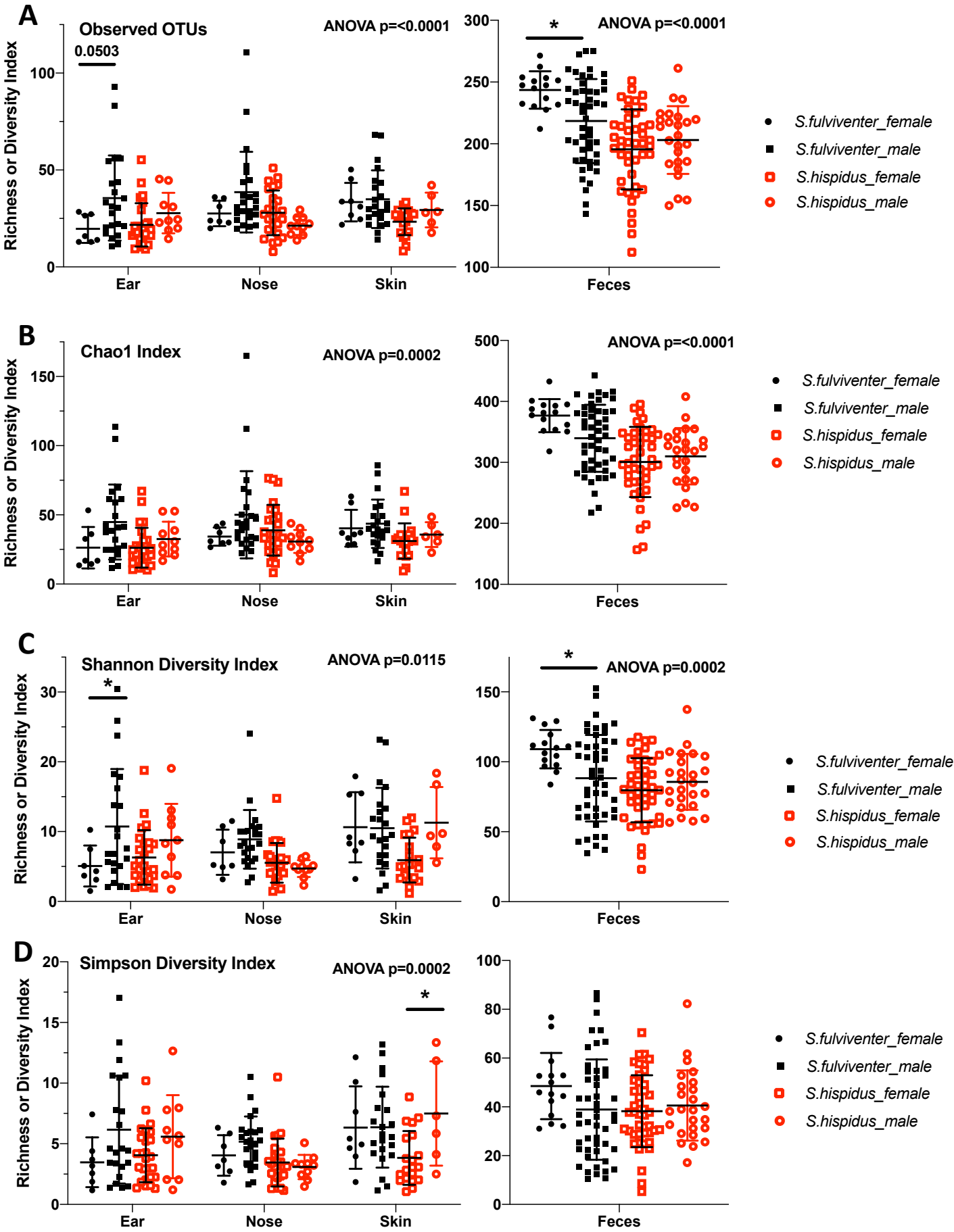

Supplement: Supplementary file 7 — Additional file 7: Figure S7. Alpha diversity metrics of ear, nose, skin, and feces between male and female S. fulviventer and S. hispidus. Richness and diversity were determined using the following methods: A) Observed OTUs, B) Chao1 index, C) Shannon Diversity Index, and D) Simpson Diversity Index. Statistical testing between gender of each cotton rat species was performed using a one-way (feces) and two-way (external sites) ANOVA, followed by a Tukey’s post-hoc test. The p-value as a result of all comparisons is shown in the top right; pairwise comparisons that were found to be significant with the Tukey post-hoc test are denoted by a bar and asterisk above the groups being compared. Statistical testing across species is not shown. Feces were plotted separately to account for the discrepancy between the Y axes. ns = P > 0.05, * = P ≤ 0.05, ** = P ≤ 0.01, *** = P ≤ 0.001, **** = P ≤ 0.0001. [file 42523_2021_90_MOESM7_ESM.pdf]

FIGURE S7

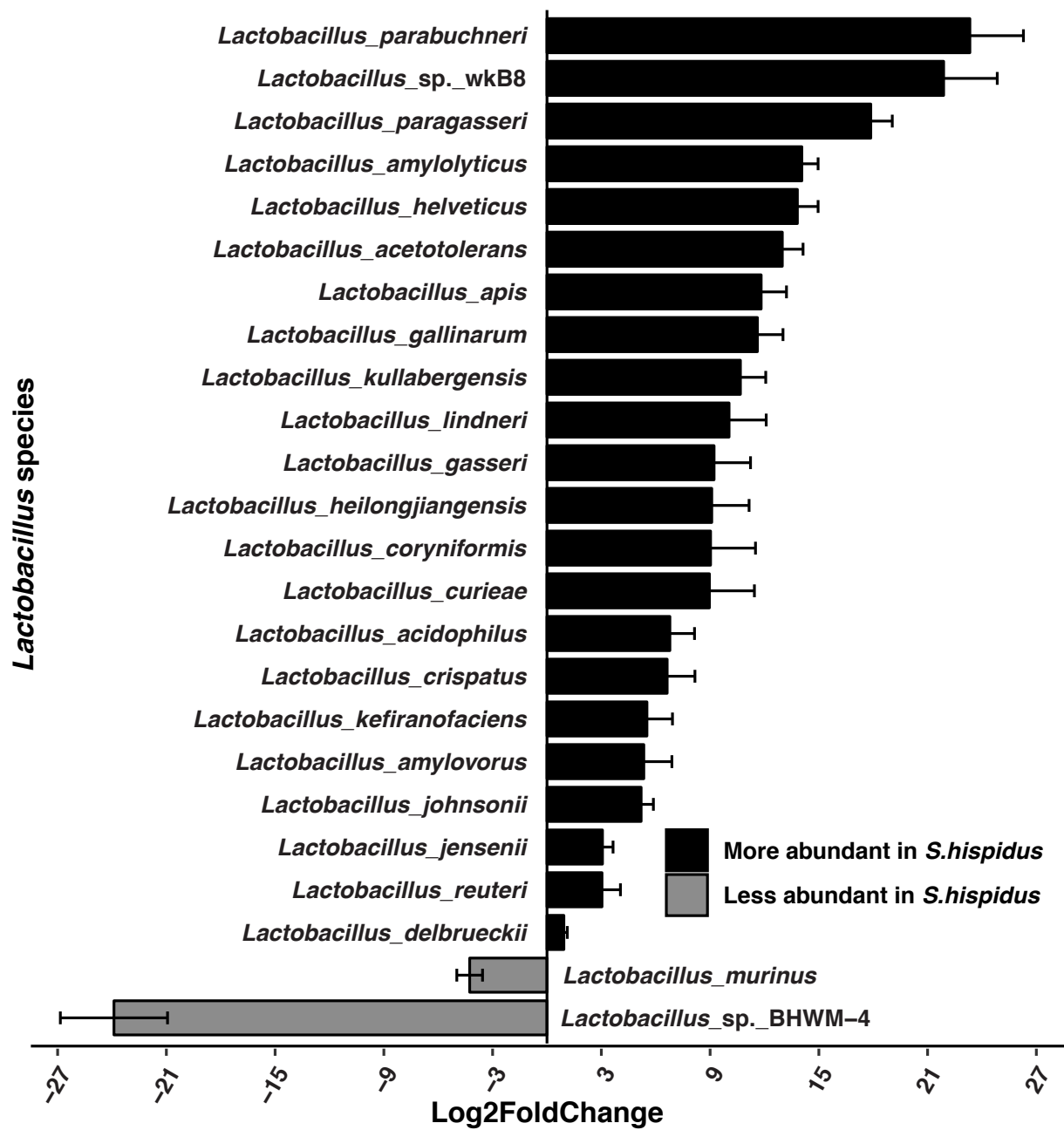

Supplement: Supplementary file 8 — Additional file 8: Figure S8. DESeq2 results representing differential abundance of Lactobacillus species and strains between S. hispidus and S. fulviventer. Data were generated from whole metagenome sequencing. [file 42523_2021_90_MOESM8_ESM.pdf]

FIGURE S8

A

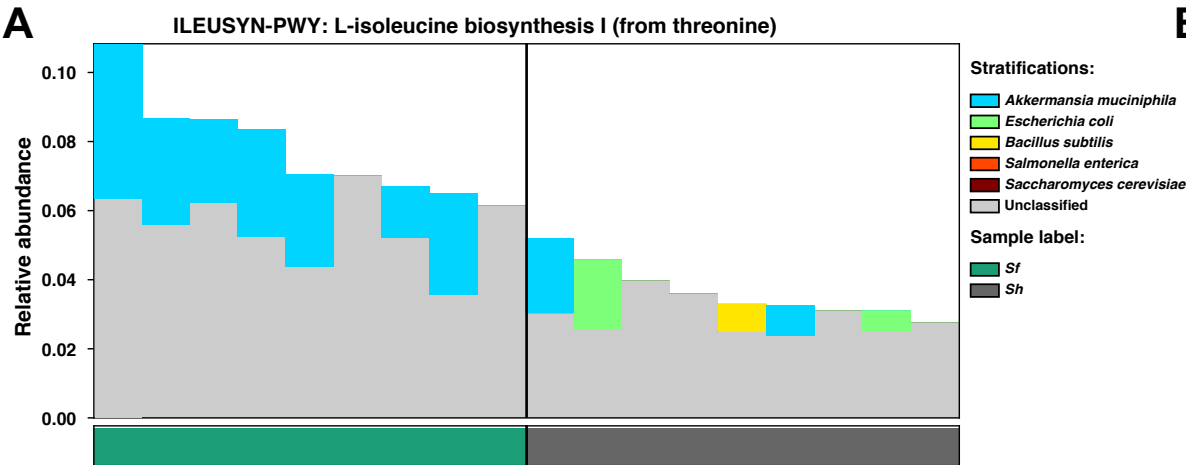

B

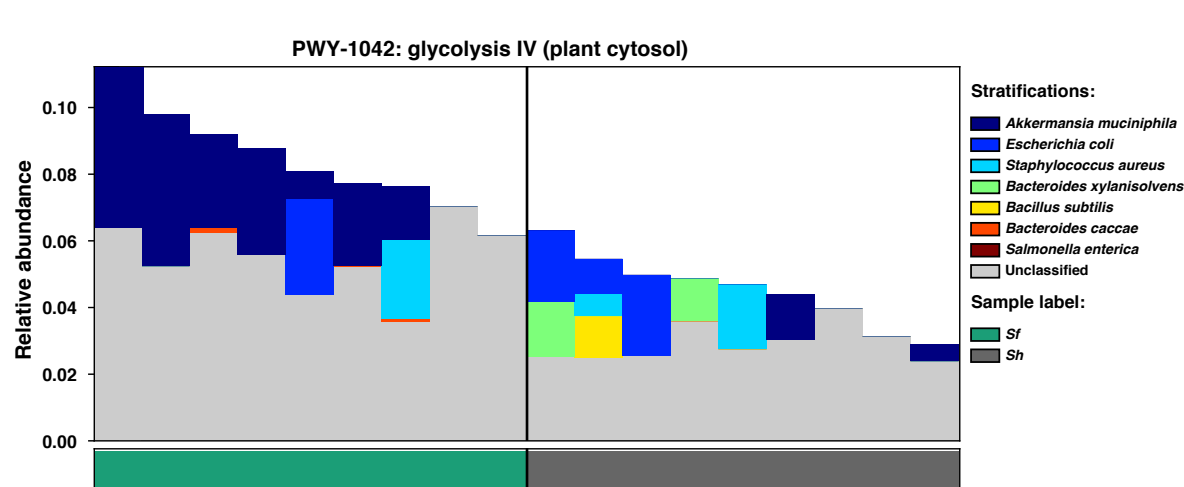

C

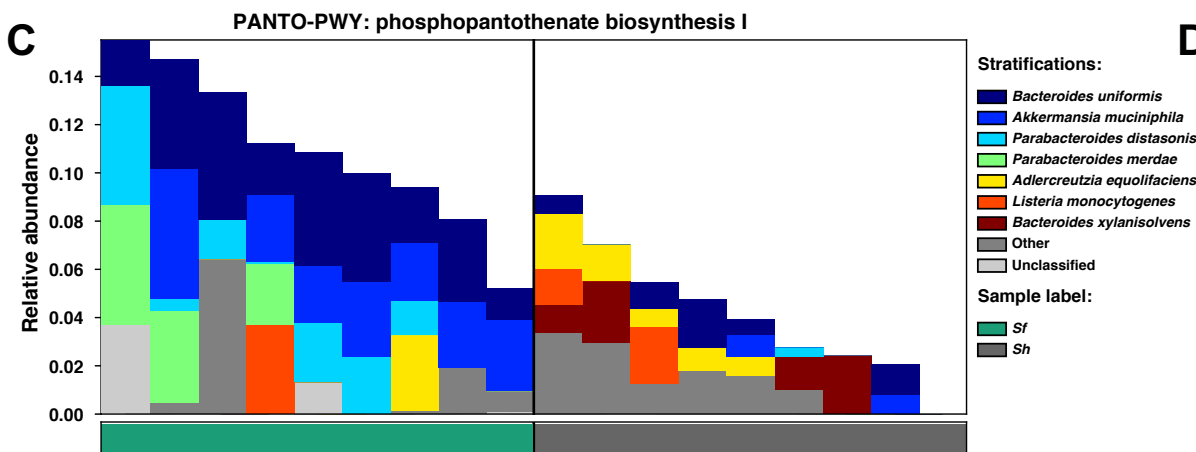

D

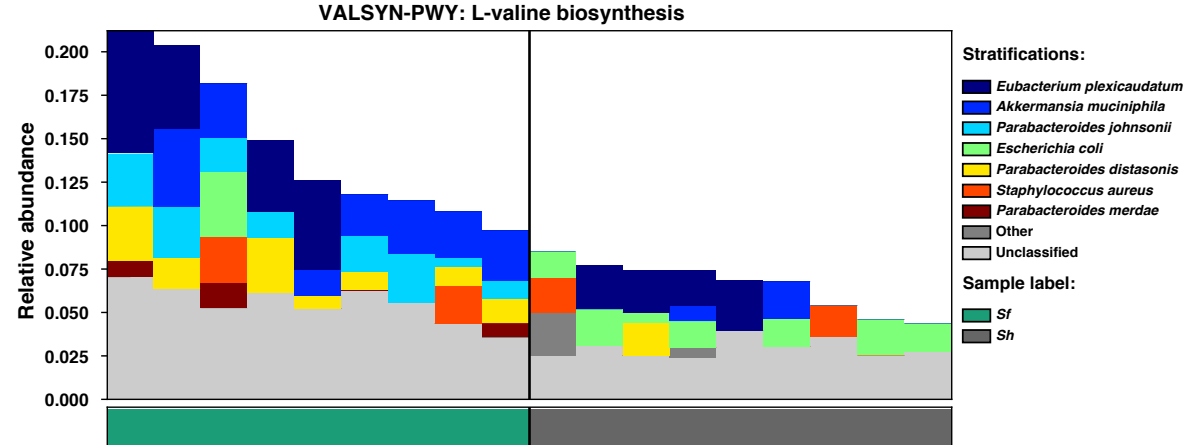

Supplement: Supplementary file 9 — Additional file 9: Figure S9. Several pathways that were more active in S. fulviventer than S. hispidus were greatly contributed to by Akkermansia species. (A) A member of the superpathway of branched chain amino acid biosynthesis, that generates not only isoleucine, but also leucine and valine (ILEUSYN-PWY). (B) Degradation of starch for the generation of carbon skeletons, reductants, and ATP for anabolic bacterial fatty acid pathway initiation via pyruvate decarboxylation to acetyl CoA (PWY-1042). (C) Biosynthesis of R-4′-phosphopantothenate, the universal precursor for the synthesis of coenzyme A and acyl carrier protein. Only plants and microorganisms can synthesize pantothenate de novo; animals require a dietary supplement. Synonymous with Vitamin B5 synthesis. (PANTO-PWY). (D) A member of the superpathway of branched chain amino acid biosynthesis, that generates not only valine, but also leucine and isoleucine. [file 42523_2021_90_MOESM9_ESM.pdf]
