# Supplementary material for: Microbial community structure and composition is associated with host species and sex in Sigmodon cotton rats
Source: Anim Microbiome. 2021 Apr 16;3:29. doi: 10.1186/s42523-021-00090-8 (PMC8051552; doi:10.1186/s42523-021-00090-8)
Supplement: Supplementary file 2 — Additional file 2: Figure S2. Top 20 most abundant bacterial genera at each body site of S. hispidus and S. fulviventer. In both species of cotton rats, external sites (skin, ear, nose) shared similar dominating genera while there were notable difference in gut taxa between S. hispidus and S. fulviventer. Not all reads were able to be classified down to the genus level; the lowest taxonomic level available is reported. The letter after the classification denotes the lowest taxonomic level able to be identified for the particular OTU (i.e., g for genus, f for family, o for order, c for class, p for phylum). [file 42523_2021_90_MOESM2_ESM.pdf]

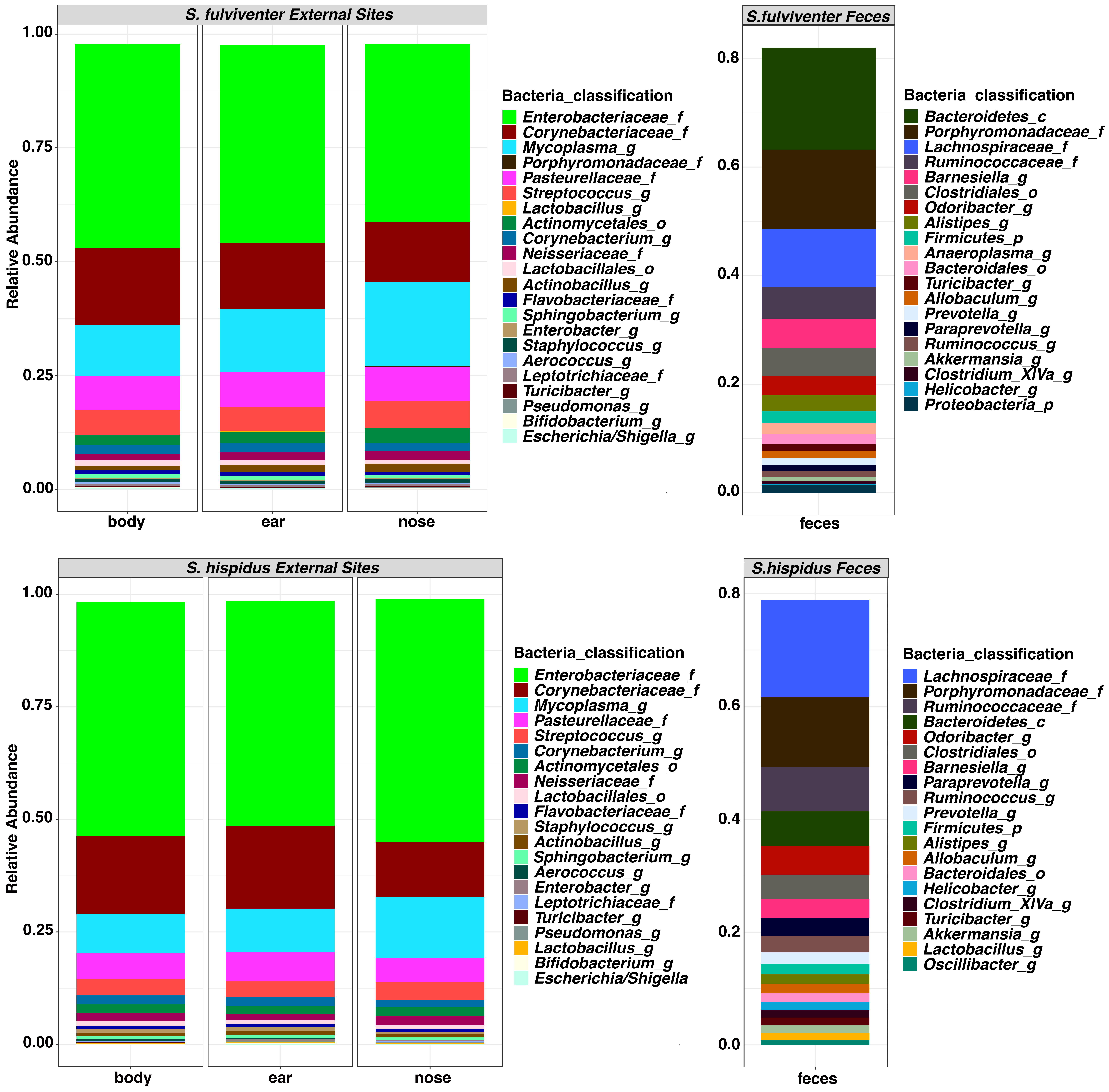

S. fulviventer External Sites
Relative Abundance
1.00
0.75
0.50
0.25
0.00
body
ear
nose
Bacteria_classification
Enterobacteriaceae_f
Corynebacteriaceae_f
Mycoplasma_g
Porphyromonadaceae_f
Pasteurellaceae_f
Streptococcus_g
Lactobacillus_g
Actinomycetales_o
Corynebacterium_g
Neisseriaceae_f
Lactobacillales_o
Actinobacillus_g
Flavobacteriaceae_f
Sphingobacterium_g
Enterobacter_g
Staphylococcus_g
Aerococcus_g
Leptotrichiaceae_f
Turicibacter_g
Pseudomonas_g
Bifidobacterium_g
Escherichia/Shigella_g
S.fulviventer Feces
0.8
0.6
0.4
0.2
0.0
feces
Bacteria_classification
Bacteroidetes_c
Porphyromonadaceae_f
Lachnospiraceae_f
Ruminococcaceae_f
Barnesiella_g
Clostridiales_o
Odoribacter_g
Alistipes_g
Firmicutes_p
Anaeroplasma_g
Bacteroidales_o
Turicibacter_g
Allobaculum_g
Prevotella_g
Paraprevotella_g
Ruminococcus_g
Akkermansia_g
Clostridium_XIVa_g
Helicobacter_g
Proteobacteria_p
S. hispidus External Sites
Relative Abundance
1.00
0.75
0.50
0.25
0.00
body
ear
nose
Bacteria_classification
Enterobacteriaceae_f
Corynebacteriaceae_f
Mycoplasma_g
Pasteurellaceae_f
Streptococcus_g
Corynebacterium_g
Actinomycetales_o
Neisseriaceae_f
Lactobacillales_o
Flavobacteriaceae_f
Staphylococcus_g
Actinobacillus_g
Sphingobacterium_g
Aerococcus_g
Enterobacter_g
Leptotrichiaceae_f
Turicibacter_g
Pseudomonas_g
Lactobacillus_g
Bifidobacterium_g
Escherichia/Shigella
S.hispidus Feces
0.8
0.6
0.4
0.2
0.0
feces
Bacteria_classification
Lachnospiraceae_f
Porphyromonadaceae_f
Ruminococcaceae_f
Bacteroidetes_c
Odoribacter_g
Clostridiales_o
Barnesiella_g
Paraprevotella_g
Ruminococcus_g
Prevotella_g
Firmicutes_p
Alistipes_g
Allobaculum_g
Bacteroidales_o
Helicobacter_g
Clostridium_XIVa_g
Turicibacter_g
Akkermansia_g
Lactobacillus_g
Oscillibacter_g
