# Supplementary material for: Microbial community structure and composition is associated with host species and sex in Sigmodon cotton rats
Source: Anim Microbiome. 2021 Apr 16;3:29. doi: 10.1186/s42523-021-00090-8 (PMC8051552; doi:10.1186/s42523-021-00090-8)
Supplement: Supplementary file 10 — Additional file 10: Table S1. Differential abundance analysis of taxa between individual body site across female and male S. hispidus and S. fulviventer. Positive Log2FoldChange = higher in females; negative Log2FoldChange higher in males. There were no significant taxa in S. fulviventer feces. [file 42523_2021_90_MOESM10_ESM.pdf]

Species/ SampleType	Bacteria (genus)	BaseMean	log2FoldChange (femaleVmale)	pvalue	qvalue
S. fulviventer Nose	*Anaerostipes*	27.559	19.968	3.53E-08	2.22E-06
	Kineococcus	17.698	19.351	9.19E-08	2.31E-06
	Comamonadaceae_unc	94.021	7.820	1.65E-03	1.73E-02
	Prevotellaceae_unc	87.015	7.711	6.92E-04	8.72E-03
	Ruminococcaceae_unc	828.485	5.317	5.00E-04	7.87E-03
	Lachnospiraceae_unc	553.467	3.228	3.90E-03	3.51E-02
	Staphylococcus	6130.222	-4.815	1.10E-07	2.31E-06
S. fulviventer Ear	*Rhizobium*	4.744	18.286	4.63E-07	2.50E-05
	Allobaculum	47.850	7.700	1.57E-04	4.23E-03
S. fulviventer Skin	*Alistipes*	74.458	22.537	1.19E-12	8.12E-11
	Hymenobacter	59.414	22.219	8.50E-10	2.89E-08
	Ruminococcaceae_unc	401.669	5.385	1.12E-03	1.90E-02
	Mucilaginibacter	50.502	-10.252	8.79E-04	1.90E-02
S. hispidus Feces	*Erysipelotrichaceae_unc*	142.661	2.677	5.71E-06	3.20E-04
	Allobaculum	5401.554	1.775	1.34E-03	2.50E-02
	Clostridium_IV	1171.401	-3.571	1.20E-03	2.50E-02
S. hispidus Nose	*Flavobacteriaceae_unc*	2853.705	2.635	4.61E-05	7.68E-04
	Betaproteobacteria_unc	63.140	2.510	4.99E-03	3.12E-02
	Neisseriaceae_unc	3774.233	2.246	1.18E-04	1.47E-03
	Mycoplasma	14945.290	1.952	2.95E-04	2.95E-03
	Streptococcus	8346.633	1.462	6.93E-03	3.85E-02
	Cellulosilyticum	72.399	-3.547	8.66E-03	4.33E-02
	Corynebacteriaceae_unc	1515.694	-4.873	3.70E-06	9.24E-05
	Alistipes	44.780	-7.300	1.78E-03	1.27E-02
	Odoribacter	39.825	-7.715	1.16E-03	9.68E-03
	Leuconostoc	34.683	-22.369	1.23E-17	6.13E-16
S. hispidus Ear	*Planococcaceae_unc*	46.965	24.491	3.42E-19	1.78E-17
	Burkholderia	76.137	6.968	1.74E-04	2.26E-03
	Ruminococcaceae_unc	36.895	-8.112	8.54E-05	1.48E-03
	Clostridium_sensu_stricto	131.715	-8.288	5.45E-06	1.42E-04
S. hispidus Skin	*Lactococcus*	73.115	5.542	1.71E-03	7.83E-03
	Turicibacter	180.005	-4.568	1.63E-03	7.83E-03
	Sphingobacterium	5341.196	-4.646	1.33E-04	1.07E-03
	Enterobacteriaceae_unc	10677.297	-5.124	5.72E-06	9.15E-05
	Flavobacteriaceae_unc	261.934	-5.649	3.78E-05	4.03E-04
	Bacteroidetes_unc	39.244	-6.347	1.18E-03	7.57E-03
	Clostridium_IV	91.701	-24.197	6.27E-15	2.01E-13

TableS1: Differential abundance analysis of taxa between individual body site across female and male *S. hispidus* and *S. fulviventer.* Positive Log2FoldChange = higher in females; negative Log2FoldChange higher in males. There were no significant taxa in *S. fulviventer* feces.
